# Crucial Gram-positive type IV secretion system protein TraF is a structural homolog of type VII secretion system protein EssB/YukC

**DOI:** 10.1093/femsml/uqag009

**Published:** 2026-03-23

**Authors:** Kirill Kuhlmann, Amrutha Stallinger, Claudia Michaelis, Tamara Margot Ismael Berger, Verena Kohler, Bernd Gesslbauer, Tea Pavkov-Keller, Elisabeth Grohmann, Walter Keller

**Affiliations:** Institute of Molecular Biosciences, University of Graz, 8010 Graz, Austria; Institute of Molecular Biosciences, University of Graz, 8010 Graz, Austria; Faculty of Life Sciences and Technology, Department of Microbiology, Berliner Hochschule für Technik, 13353 Berlin, Germany; Institute of Molecular Biosciences, University of Graz, 8010 Graz, Austria; Institute of Molecular Biosciences, University of Graz, 8010 Graz, Austria; Department of Molecular Biology, Umeå University, 901 87 Umea, Sweden; Umeå Centre for Microbial Research, Umeå University, 901 87 Umeå, Sweden; Institute of Pharmaceutical Sciences, Division of Pharmaceutical Chemistry, University of Graz, 8010 Graz, Austria; Institute of Molecular Biosciences, University of Graz, 8010 Graz, Austria; BioTechMed Graz, 8010 Graz, Austria; Field of Excellence BioHealth - University of Graz, 8010 Graz, Austria; Faculty of Life Sciences and Technology, Department of Microbiology, Berliner Hochschule für Technik, 13353 Berlin, Germany; Institute of Molecular Biosciences, University of Graz, 8010 Graz, Austria; BioTechMed Graz, 8010 Graz, Austria; Field of Excellence BioHealth - University of Graz, 8010 Graz, Austria

**Keywords:** type IV secretion, bacterial conjugation, pIP501, pseudokinase, type VII secretion, *Enterococcus faecalis*, multiresistant pathogens

## Abstract

Type IV secretion systems (T4SS) are found in both monoderm and diderm bacteria. The broad-host-range conjugative plasmid pIP501 from *Enterococcus faecalis* harbors a T4SS encoding 15 *tra* genes responsible for the spread of antimicrobial resistance genes among diverse G+ pathogens. Eight Tra proteins (TraB, TraC_B3_, TraF, TraH_B8_, TraI, TraK, TraL_B6_, and TraM_B8_) are postulated to form the mating pair formation (MPF) complex representing the central DNA translocation pore. One of these proteins is TraF, a 52.8 kDa transmembrane protein, which lacks any homologs in other well described T4SSs. In this study, TraF was proven to be an essential conjugative transfer protein. The TraF pulldown co-eluted all Tra proteins except TraG_B1_ and TraN. Bacterial-two-hybrid assay showed a strong interaction between TraF and TraM_B8_. We present a 1.25 Å resolution crystal structure of the N-terminal domain of TraF, which adopts a pseudokinase fold. AlphaFold predictions of full-length TraF with membrane mimetics show a transmembrane protein with two distinct soluble domains. FoldSeek revealed a strong similarity to YukC (EssB), a transmembrane pseudokinase from type VII secretion system (T7SS). YukC was shown to function as an interaction hub by mediating contacts between its pseudokinase domain and other T7SS proteins as part of the central membrane core complex. We postulate that TraF might play an important role in T4SS complex formation.

## Teaser

Crystal structure of the soluble domain of TraF, a transmembrane T4SS protein, reveals structural homology to the T7SS protein YukC/EssB.

## Introduction

Currently, humanity is facing an antibiotic resistance crisis as many pathogens have acquired resistance against multiple antibiotic classes. The so-called multidrug-resistant bacteria (MDR) developed several strategies to diminish or eliminate the efficacy of drugs (Ho et al. 2025). Resistance mechanisms can be acquired either by the uptake of plasmids or encoded on the chromosome, e.g. on ICEs (integrative conjugative elements) and IMEs (integrative mobilizable elements) (Libante et al. 2020, Botelho and Schulenburg 2021, Gomberg and Grossman 2024, Garcillán-Barcia et al. 2025, Kumavath et al. 2025) The number of pathogens with resistance against multiple antibiotic classes has increased tremendously over the past few years. It is estimated that in 2021 antimicrobial resistant (AMR) bacteria were associated with 4.71 million deaths and forecasted to culminate in ∼10 million deaths annually by 2050 (UN environment programme 2023, Naghavi et al. 2024, WHO 2025). In 2024, WHO updated its bacterial priority pathogen list (BPPL) which categorizes pathogens into priority groups to combat AMRs. *Enterococci*, specifically *E. faecium* have been classified as high-priority pathogens due to their ability to transmit antibiotic resistance genes across the One Health spectrum (WHO 2024).

There are three main horizontal transfer mechanisms by which bacteria take up new genes: through transformation, transduction, or conjugative transfer.

The latter mechanism, mediated by T4SS, can involve either the transfer of plasmids, ICEs, or genomic islands. T4SSs are better characterized in G- bacteria, where structures of T4SS complexes in different compositions have been solved.

As recently reviewed, multiple cryo-EM structures of outer membrane complexes (OMC) and T4SS assemblies of increasing complexity from G- bacteria have been solved (Costa et al. 2024, Paillard et al. 2025, Waksman 2025).

Gram-negative (diderm) bacteria possess a thin monolayered peptidoglycan with an outer membrane, meanwhile the gram-positive (monoderm) bacteria solely feature a thick multilayered peptidoglycan resulting in pronounced structural differences of their envelopes. In addition, lipid composition as well as the presence of lipopolysaccharides (LPS) in diderms distinguishes them from monoderm bacteria which possess (lipo)teichoic acids (LTAs and TAs) instead (Malanovic and Lohner 2016).

Therefore, the architecture of T4SSs also differs: diderm bacteria T4SS proteins assemble into an ATP energy center attached to an inner membrane complex (IMC) as well as an outer membrane core complex (OMCC) and a conjugative pilus (Costa et al. 2021, Christie et al. 2026). From monoderm systems, structural information has only been obtained for separate proteins with a reconstruction of a T4SS complex obtained by modeling showing a large single-membrane-spanning complex which lacks the OMCC and pilus (Breidenstein et al. 2025).

An important plasmid model to study horizontal gene transfer (HGT) in monoderm bacteria is the broad-host-range Inc18 plasmid pIP501 encoding a T4SS used by a variety of G+ bacteria (Kohler et al. 2018). Inc18 plasmids are often harbored by biofilm producing bacteria frequently living in communities of multiple bacterial strains (Michaelis and Grohmann 2023). The close cell-to-cell contact in such biofilms increases the risk of interspecies transfer.

Despite the differences in the cell envelope architecture, T4SSs are found in both monoderm and diderm bacteria with various protein members sharing analogy between the systems. In the pIP501 T4SS, TraH (TraH_B8_) and the DNA binding protein TraM (TraM_B8_) were characterized as VirB8 homolog, TraG (TraG_B1_), a lytic transglycosylase and endopeptidase, was identified as a VirB1 homolog (Arends et al. 2013). It interacts with the MPF complex protein TraM_B8_ and is crucial for its correct insertion into the cell membrane (Kohler et al. 2017). TraE (TraE_B4_) was identified as a VirB4-like ATPase meanwhile TraI likely acts as a membrane anchoring factor for the VirD4-like coupling protein TraJ (TraJ_D4_) (Grohmann et al. 2016). TraL (TraL_B6_) is a VirB6-homolog (Breidenstein et al. 2025).

Besides HGT, T4SSs are responsible for intercellular transport of virulence factors in both monoderm and diderm organisms, whereas T7SSs are solely mediating effector transport across cell envelopes of pathogenic mycobacteria (T7SSa) and some bacillota such as *Staphylococcus aureus* and *Bacillus subtilis* (T7SSb) (Christie et al. 2014, Costa et al. 2024, Garrett et al. 2024). T7SSa encode membrane-bound components such as EccB, the AAA+ ATPAse EccC, EccD, EccE and MycP as well as the cytoplasmic ATPase EccA (EccE and EccA are missing in some ESX-4 systems). T7SSb are distantly related to T7SSa. They feature a different set of components including membrane-proteins EsaA, EssA, EssB, and EssC (EccC analog) as well as cytosolic components EsaE and EsaB (Bowman and Palmer 2021, Garrett et al. 2024).

In this study, we aimed to elucidate the function and interaction partners of the putative MPF complex factor TraF_pIP501_. The protein was demonstrated to be essential for conjugative transfer. To the best of our knowledge, TraF is the first conjugative T4SS protein showing structural similarities to pseudokinases from monoderm T7SSs involved in effector secretion, like EssB from *Geobacillus thermodenitrificans* (Zoltner et al. 2013b, Bowman and Palmer 2021), EssB from *S. aureus* (Zoltner et al. 2013a, Bowman and Palmer 2021) and YukC from *B. subtilis* (Tassinari et al. 2022, Oka et al. 2025).

## Materials and methods

### Computational analysis of TraF

The different domains of TraF were identified using PSIPRED (Jones 1999). The transmembrane segment of the linker helix was determined with the online server DeepTMHMM (Hallgren et al. 2022). The 3-D structure of the full-length TraF and its C-terminal domain (TraF_239-450_) were predicted by AlphaFold3 accessed through the AlphaFold Server by Google LLC (https://alphafoldserver.com/) as well as through a local AlphaFold3 installation (https://github.com/google-deepmind/alphafold3). A simulation of a lipid environment was achieved on the local AlphaFold3 installation by adding CCD parameterized phosphatidyl-glycol representatives (PubChem CID: 44 566 653) as well as cardiolipins (PubChem CID: 5 287 898, ePDB ID: CDL) to the prediction input. CCD compliant cif files were generated from sdf coordinates using a custom python script utilizing rdkit (https://github.com/rdkit/rdkit). The predicted alignment error (PAE) matrix was generated using “PAE Viewer” web server (Elfmann and Stülke 2023). All atom and Cα-Cα alignment of structures was performed using PyMOL (v 2.5.4, Schrödinger LLC) “super” method with outlier rejection: cutoff 2, cycles 5 and “cealign” method respectively. Foldseek (Van Kempen et al. 2024) server was used to search for structural homologs.

### Construction of a *traF* in-frame deletion mutant of pIP501

All strains, plasmids and primers used in this work are listed in Supplementary Table S1 and S2. The pIP501∆*traF* in-frame deletion mutant was constructed in *Enterococcus faecalis* JH2-2 using a previously described marker-less allelic exchange strategy (Kristich et al. 2007). The *traF* upstream and downstream flanking regions (1165 bp and 899 bp, respectively) were amplified using *E. faecalis* JH2-2 (pIP501) as DNA template. Both fragments were subcloned into pUC18 using the corresponding restriction sites, PstI/XbaI and BamHI/EcoRI, to create pUC18-UPS-DWS-*traF*. More than 98% of the *traF* coding region was deleted, retaining only three intact N-terminal and four C-terminal codons of *traF* to prevent polar effects on downstream *tra* genes. The fused UPS-DWS fragment was excised and inserted into the suicide plasmid pKA using the PstI and EcoRI restriction sites followed by transformation into *E. coli* EC1000 (Addgene, Watertown, MA, USA). To obtain the pIP501∆*traF* mutant, the protocol as described in Berger et al. (2022) was followed. The correctness of the deletion mutant was confirmed by sequencing the deletion borders.

### Complementation of *E. faecalis* JH2-2 (pIP501∆*traF*)

To restore pIP501 transfer in the pIP501∆*traF* knockout (KO) strain, the *traF* wild-type (WT) gene with its ribosomal binding site (RBS) was amplified from pIP501 using primers containing BstYI/SalI restriction sites and inserted into the shuttle vector pEU327, resulting in pEU327-RBS-*traF*.

For *traF* KO complementation, the *traF* WT gene with its ribosomal binding site (RBS) was amplified from pIP501 with primers containing BstYI/SalI restriction sites and inserted into the shuttle vector pEU327 followed by transformation into *E. coli*.

As a control for the complementation experiments, the full length TraF (*traF*) and TraF with a C-terminal Strep-tag (*traF-*Strep) was used. Full-length *traF* was amplified from pIP501 with primers containing BstYI/SalI restriction sites. For amplification of pEU327-Strep, GA_pEU327_C Strep fw and GA_pEU327_C Strep rev primers were used. To incorporate the *traF* gene with its native RBS into pEU327-Strep, a Gibson Assembly Kit (New England Biolabs, Ipswich, MA, USA) and primers GA_RBS-traF fw and GA_RBS-traF rev were applied according to the manufacturer’s manual resulting in pEU327-RBS-*traF* with a C-terminal Strep-tag (pEU327-RBS-*traF*-Strep). For the partial complementation of pIP501∆*traF*, four pEU327 plasmids were constructed. They either contain the sequence of the (1) N-terminal domain (NTD) (*traF*_1-212_), (2) C-terminal domain (CTD) (*traF*_239-450_), and combinations of (3) NTD and transmembrane helix domain (TMD) (*traF*_1-238_), or (4) a segment containing NTD, TMD and CTD (*traF*_176-450_).

To obtain plasmids pEU327-RBS-*traF*_1-212_, pEU327-RBS-*traF*_1-238_, and pEU327-RBS-*traF*_239-450_, the respective *traF* fragments were generated as described for full-length *traF* above using pEU327_BstYI_traF fw with pEU327_SalI_traF1-212 rev and pEU327_SalI_traF1-238 rev as well as pEU327_BstYI_traF239-450F primer.

The plasmid pEU327-RBS-*traF*_176-450_ was constructed using site-directed mutagenesis (Q5 site-directed mutagenesis kit, New England Biolabs, Ipswich, MA, USA) by introducing a deletion of *traF*_2-175_ into the plasmid pEU327-RBS-*traF* with the primers Mut_traF176-450 fw and Mut_traF176-450 rev.

To study the replaceability of the TMD_TraF_ with a TMD of another pIP501-encoded T4SS Tra protein of the same length, the TMD sequence of *traF* (*traF*_215-234_) was substituted for the TMD sequence of *traB* (*traB*_45-64_). The plasmid pEU327-RBS-*traF* served as a template for Q5 site-directed mutagenesis using the primers Mut_traF_TMD1-B fw and Mut_traF_TMD1-B rev to get pEU327-RBS-*traF:: TMD_traB_*.

In addition to complementation with truncated TraF variants, we tested whether independent expression of NTD (TraF_1-212_), TMD, and CTD (TraF_213-450_) could restore the transfer of pIP501∆*traF*. We used pEU327 for tandem expression of the domains under control of the *xylA* promoter by introducing the following features between TraF aa 212 and 213: two-stop codons, a 45-bp spacer region (randomized order of Leu, Ala, Gly codons), RBS*_traF_*, a 6-bp linker sequence, and a start codon for the CTD*_traF_*. The plasmid was named pEU327-RBS-NTD*_traF_*-RBS-TMD*_traF_*-CTD*_traF_*.

For biparental mating assays, *E. faecalis* JH2-2 (pIP501∆*traF*) was electroporated with one of the eight pEU327-RBS-*traF* variants (*traF, traF-Strep*, pEU327-RBS-*traF::TMD_traB_, traF*_1-212_, *traF*_1-238_, *traF*_239-450_, *traF*_176-450_, RBS-NTD*_traF_*-RBS-TMD*_traF_*-CTD_t_*_raF_*).

*In vivo* expression of TraF variants, TraF-N, TraF-N+TMD_TraF_, TraF-C, TraF::TMD_TraB_, and TraF-C+TMD_TraF_ was verified in *E. faecalis* JH2-2 by immunoblotting. Full-length TraF-Strep was used as positive control. As the anti-TraF antibody was raised against TraF-C, TraF-N and TraF-N+TMD_TraF_ cannot be detected.

### Biparental mating assay

Biparental mating assays were performed as previously described in Berger et al. (2022). Following donor strains were used: the isogenic *E. faecalis* (pIP501), *E. faecalis* (pIP501∆*traF*), *E. faecalis* (pIP501∆*traF*, pEU327-RBS-*traF*), *E. faecalis* (pIP501∆*traF*, pEU327-RBS-*traF*-Strep), *E. faecalis* (pIP501∆*traF*, pEU327-RBS-*traF*_1-212_), *E. faecalis* (pIP501∆*traF*, pEU327-RBS-*traF*_1-238_), *E. faecalis* (pIP501∆*traF*, pEU327-RBS-*traF*_176-450_), *E. faecalis* (pIP501∆*traF*, pEU327-RBS-*traF*_239-450_), *E. faecalis* (pIP501∆*traF*, pEU327-RBS-*traF::TMD_traB_*), and *E. faecalis* (pIP501∆*traF*, pEU327-RBS-NTD*_traF_*-RBS-TMD*_traF_-*CTD*_traF_*). Plasmid-free *E. faecalis* OG1X was used as the recipient strain. All mating assays were performed as triplicates. Transfer rates (number of transconjugants per recipient cell) are given with standard deviations. Significance and *p-*values were calculated using the Mann-Whitney U test. Significance is indicated by asterisks. ** *P* < 0.01.

### Bacterial-2-hybrid assay

The Bacterial-2-hybrid (B2H)-assay was performed as described in Kohler et al. (2017) with some modifications. In this study, the plasmids pKT25, pKNT25, pUT18, and pUT18C harboring the *tra* genes *traF, traG, traJ, traL, traM* were transformed into *E. coli* BTH101 and grown on LB agar plates supplemented with respective antibiotic (100 µg ml^−1^ ampicillin (Amp) for pUT18 and pUT18C, 50 µg ml^−1^ kanamycin (Kan) for pKT25 and pKNT25) overnight (ON) at 37°C at 1000 rpm. The main culture was inoculated with the overnight culture (ONC) and induced with IPTG after 4 h at 30°C followed by a β-galactosidase assay adapted from (Griffith and Wolf 2002). The GCN4 leucine zipper fused to T18 or T25 fragments, was used as positive interaction control. The empty vector in combination with different vector variants (either empty T18/T25 fragment or fused to *traF, traG, traJ, traL*, and *traM*) was utilized as negative controls. All values were normalized to the positive control. The results represent the mean of six measurements (*n* = 6) for TraM_B8_-TraM_B8_, TraF-TraF, and TraF-TraM_B8_, four measurements for TraM_B8_-TraF interaction (*n* = 4), between ten and 15 measurements for TraG_B1_-TraM_B8_, TraJ_D4_-TraM_B8_, and TraL_B6_-TraM_B8_ (*n* = 10–15).

### Construction of plasmids used in pull-down assays

The TraF-Strep pulldown construct (pRBBm59-RBS-*traF*-Strep) was generated from pRBBm59-RBS-*traJ-*Strep by replacing the *traJ* gene with *traF. traF* was amplified from pIP501 with fw_TraF_pRBBm59 and rev_TraF_pRBBm59 primers and cloned into pRBBm59-RBS-*traJ*-Strep using BamHI and NotI restriction sites replacing *traJ*. The *E. coli*/*Priestia megaterium* (previously known as *Bacillus megaterium*) shuttle vector pRBBm59-RBS-*gfp*-Strep was used as negative control as described in Berger et al. (2022). *Priestia megaterium* MS941 (pMGBm19-RBS-*traB*-*traO*) was transformed with pRBBm59-RBS-*traF*-Strep according to the protocol of Biedendieck et al. (2011). Transformants were selected on LB agar containing 35 µg ml^−1^ chloramphenicol (Cam) and 10 µg ml^−1^ tetracycline (Tet) and were screened for the presence of the respective pRBBm59 plasmids by colony PCR.

### Pull down assay—isolation of potential TraF interaction partners

Expression and purification of Strep-tagged TraF and its interaction partners from *P. megaterium* MS941 (pMGBm19-RBS-*traB*-*traO*, pRBBm59-RBS-*traF*-Strep) were performed as described by (Berger et al. 2022) and (Michaelis et al. 2024) with some modifications.

The expression cultures (1.2 l in 5 l baffled flasks) were inoculated to an OD_600_ of 0.01 and incubated under constant shaking at 37°C until an OD_600_ of 0.3–0.4. The expression of TraB-TraO and Strep-tagged TraF was induced by the addition of 0.5% (w/v) xylose for pMGBm19 and 0.5% (w/v) sucrose for pRBBm59. After 6 h, the cells were harvested by centrifugation at 9000 × g at 4°C for 60 min. The cell pellet was resuspended in thrice the cell wet weight of complex binding buffer (100 mM Tris/HCl, 300 mM NaCl, 1 mM EDTA, pH 8.0), supplemented with protease inhibitor (1 µg ml^−1^ pepstatin, 2 µg ml^−1^ antipain, 20 µg ml^−1^ leupeptin in DMSO). The cells were lysed by sonication for 1 h on ice [50% duty cycle, 60% intensity; Sonopuls, UW/HD 2070, (Bandelin electronic GmbH & Co. KG, Germany)]. After 60 min of centrifugation at 4°C and 38 000 × g, the supernatant was filtered through a MF-Millipore 0.45 µm MCE membrane (Merck, Darmstadt, Germany) to eliminate residual cell debris. The filtered lysate was loaded onto a 5 ml StrepTrap^TM^ HP column (Cytiva, Marlborough, Massachusetts, USA) pre-equilibrated with complex binding buffer using an ÄKTA pure 25-l chromatography system (Cytiva). The proteins were eluted using the complex binding buffer supplemented with 2.5 mM desthiobiotin and the peak fraction processed by mass spectrometry as described in “Mass spectrometry analysis on TraF pulldown”.

### Cloning of the TraF domains

The soluble domains of TraF, TraF-N (Met1-Arg212), and TraF-C (Glu239-Asp450), were cloned into the expression plasmid pQTEV using BamHI and KpnI sites. The resulting constructs consist of an N-terminal 7x-His-tag followed by a tobacco etch virus (TEV) protease cleavage site and the respective soluble domain of TraF. The recombinant vectors were transformed into competent *E. coli* BL21-CodonPlus (DE3) cells (Stratagene, Amsterdam, The Netherlands). For selenomethionine (SeMet) expression, *E. coli* B834 (DE3) (Merck) cells were used.

### Heterologous expression and purification of TraF-N and TraF-C

Expression was performed in 500 ml LB medium. BL21 CodonPlus cells were cultured in the presence of 100 µg ml^−1^ Amp and 100 µg ml^−1^ Cam. The cells were grown at 37°C with continuous agitation until an OD_600_ of 0.7. After reducing the temperature to 16°C, protein expression was induced using 0.5 mM IPTG and expression continued ON. Cells were harvested by centrifugation at 3993 × g at 4°C for 20 min and immediately frozen at –20°C.

For SeMet expression in *E. coli* B834(DE3) cells, 100 µg ml^−1^ Amp was added to the medium. The cells were harvested when the OD_600_ reached ∼ 0.6. The pellets were resuspended in M9 minimal medium, and growth continued for 1 h at 37°C. The temperature was reduced to 16°C and 0.5 mM IPTG was added for induction. Additionally, 25 mg of SeMet (Sigma–Aldrich, KGaA, Darmstadt, Germany) were added to the culture, and expression proceeded ON. The cells were harvested and immediately frozen at–20°C. Expression and purification were monitored by SDS-PAGE.

Purification of TraF-N, SeMet-TraF-N, and TraF-C was carried out using the extraction buffer [50 mM Hepes, 300 mM NaCl, 1 mM phenylmethylsulfonyl fluoride (PMSF), and 1 mM benzamidine, pH 7.0]. This cell suspension was homogenized (UltraTurrax, IKA, Staufen, Germany), followed by sonication (Sonopuls HD2070, Bandelin, Berlin, Germany; 25 min, Pulse 5, 55% amplitude). Lysates were centrifuged at 16 000 × g for 30 min at 4°C.

The supernatant was filtered through 0.45 µm filters and loaded onto a HisTrap FF 5 ml column (Cytiva) for affinity purification. Fractions containing least impurities were pooled and subjected to ON dialysis, using a dialysis tube with a cutoff of 14.5 kDa (Spectra/Por® 3, Repligen, Waltheim, Massachusetts, USA), and TEV cleavage at 4°C. The cleaved protein sample was loaded onto a HisTrap FF 5 ml column to separate it from uncleaved protein, His-tag, and TEV protease. The flow-through was concentrated using Amicon Ultra Centrifugal Filters with a 3 kDa MWCO (Merck) and loaded onto a size exclusion chromatography (SEC) column (HiLoad 16/60 Superdex 200, Cytiva) as the final purification step using SEC buffer (50 mM Hepes, 150 mM NaCl, pH7.0). The purification chromatograms are shown in Supplementary Fig. S1 and S2.

### Expression studies on transcriptional level

To determine whether the KO of *traF* influences the expression level of other *tra* genes within the pIP501 operon, RNA isolation, reverse transcription (RT) and qPCR were utilized to compare gene expression of the WT strain *E. faecalis* JH2-2 (pIP501) with that of the *traF* KO strain, *E. faecalis* JH2-2 pIP501Δ*traF*.

ON cultures in BHI medium were supplemented with Cam (35 μg ml^−1^) and incubated for 16 h at 37°C. 20 ml BHI medium supplemented with Cam were inoculated with the ONC to an OD_600_ of 0.05 and incubated for 3 h. Cells equivalent to an OD_600_ of 2 were harvested by centrifugation (17 696 × g, 1 min, RT), shock-frozen in liquid nitrogen and stored at −80°C for further application.

For RNA isolation, two pellets of each sample were resuspended in 500 μl Trizole (ThermoFisher, Waltham, Massachusetts, USA) and transferred to tubes containing ∼200 μl glass beads (500 μm diameter). The cells were disrupted using a Mini Bead Beater (Biospec, Bartlesville, OK, USA) and shaken three times for 1 min at 3000 rpm. After incubation for 5 min at RT, 100 μl of 1– bromo–3–chloropropane was added, and the tubes were shaken vigorously for 15 s. After incubation for 2–3 min at RT, the cell debris was removed by centrifugation for 15 min at 4°C and 13 400 × g. Two hundred fifty μl 100% isopropanol was added to the upper phase containing RNA followed by incubation for 30 min at RT.

The samples were centrifuged for 10 min at 4°C and 13 400 × g, the pellets were washed with 500 μl of ice-cold 75% ethanol followed by centrifugation for 5 min at 4°C and 5100 × g. The pellets were air-dried for 5–10 min, resuspended in 25 μl RNAse-free water and incubated for 10 min at 55°C. The RNA concentration and integrity were determined by UV absorption and confirmed by gel electrophoresis. Contaminating DNA was removed using the DNA-free™ DNA Removal kit from ThermoFisher. For reverse transcription, the M-MLV Reverse Transcriptase RNase H- kit from Solis Biodyne (Tartu, Estonia) was used according to the manual.

To compare the gene expression of the two strains, quantitative RT qPCR was performed for distinct *tra* genes (*traB, traE, traG, traJ, traK, traM, traO*). To apply the ΔΔC_T_ method (Schmittgen and Livak 2008), *proC* was used as a housekeeping gene. 16S rRNA and *gapdh* genes were shown as additional controls. The reactions were performed in triplicates, including no-template control. The qPCR program was performed including the following steps: initial activation: 95°C, 12 min (1 cycle), denaturation: 95°C, 15 s, annealing: 65°C, 20 s, elongation: 72°C, 20 s (40 cycles).

### Protein expression studies

The effect of *traF* deletion on other *tra* genes was also studied on the protein level. Protein expression levels of *E. faecalis* JH2-2 lacking pIP501, *E. faecalis* JH2-2 pIP501, and the *traF* KO strain, *E. faecalis* JH2-2 pIP501*ΔtraF*, were compared by quantitative western blots.

To this end, the *E. faecalis* strains (JH2-2, JH2-2 pIP501, JH2-2 pIP501*ΔtraF*) were cultivated in BHI medium supplemented with 35 µg ml^−1^ Cam at 37°C under shaking. Based on the measured OD_600_, a volume of cells corresponding to an OD_600_ = 10 was harvested by centrifugation for 15 min at 8000 × g, 4°C and stored at −20°C until usage. The cell pellet was resuspended in 500 μl 1x Tris-buffered saline (TBS) buffer and disrupted by sonication (60% power, 30 s, 4°C). The samples were prepared for SDS-PAGE as per standard protocol. The protein lysates were loaded onto a 12.5% SDS polyacrylamide gel. The western blots were first probed with polyclonal anti-GroEL antibody (Sigma-Aldrich KGaA) at 1:10 000 dilution and secondary IgG antibody (Promega, Madison, WI, USA) at a dilution of 1:10 000 followed by respective polyclonal anti-Tra antibodies (anti- TraH_B8_, TraJ_D4_, TraK, TraF, and TraM_B8_) (Biogenes GmbH, Berlin, Germany) at 1:10 000 dilution and secondary horseradish peroxidase conjugated IgG antibody (Promega) at a dilution of 1:10 000. Protein expression was quantified using Biorad Image Lab 5.0 software (Bio-Rad, Hercules, California, USA) by utilizing GroEL as loading control.

### Analytical size exclusion chromatography

The oligomerization state of TraF-N and TraF-C was determined by subjecting the proteins to an analytical SEC on a Superdex 200 Increase 10/300 GL column (Cytiva), using a gel filtration standard (Bio-Rad, #1 511 901) to determine the apparent molecular weight.

### Secondary structure characterization and thermal stability

Circular dichroism (CD) measurements were carried out using a Jasco J715 spectropolarimeter (JASCO Inst., Pfungstadt, Germany) equipped with an external thermostat. Spectra were recorded from 250 nm to 190 nm in a 0.02 cm cuvette as the average of ten repeat scans. Temperature scans were performed from 25°C to 95°C with a scan rate of 1°C·min^−1^ at 208 nm. Individual spectra were recorded with 3 repeat scans from 250 nm to 190 nm for every 10°C. The data was normalized and evaluated using the DichroWeb online server (now accesible under https://github.com/pcddb/DichroWebGit) applying the CDSSTR algorithm with reference dataset #4 (Whitmore and Wallace 2004).

### Crystallization of TraF-N

The Oryx8 robot (Douglas Instruments, East Garston, Hungerford, Berkshire, UK) was used for crystallization experiments using the vapor diffusion method. Crystals were obtained for both native and SeMet labeled His-tagged TraF-N under various conditions of PEG/Ion screen (4% v/v Tacsimate™ pH 4.0, 12% w/v Polyethylene glycol 3 350) (Hampton Research, Aliso Viejo, CA, USA) and JCSG+ screen (0.2 M Sodium chloride 0.1 M Phosphate/citrate buffer pH 4.2 20% w/v PEG 8000) (Molecular Dimensions Ltd., Rotherham, UK) with 1:1 ratio of protein (7 mg ml^−1^) to precipitant solution.

### Data collection, processing and refinement

Diffraction data were collected at beamline P11 (DESY, Hamburg, Germany) (Burkhardt et al. 2016). A fluorescence scan was done to validate the presence of selenium in the TraF-N SeMet-labelled crystals. Datasets were processed using XDS (Kabsch 2010) and AutoSol (Terwilliger et al. 2009) was used to find the selenium sites. The initial model was generated using ARP/wRAP and BUCCANEER (Cowtan 2006). Refinement and interactive model building were performed with PHENIX 1.16_3594 (Adams et al. 2010) and COOT (Emsley and Cowtan 2004, Emsley et al. 2010).

For the high-resolution native data set (1.25 Å), the structure was solved by molecular replacement using PHASER MR (McCoy et al. 2007) and the SeMet structure served as a search template. The structure was rebuilt in COOT and refined using PHENIX; quality was assessed with Molprobity (Chen et al. 2010). The final high-resolution crystal structure of TraF-N (TraF_1-194_) was deposited in the protein databank (PDB) under the accession code pdb_00008cba. Figures were prepared using UCSF ChimeraX, developed by the Resource for Biocomputing, Visualization, and Informatics at the University of California, San Francisco, with support from National Institutes of Health R01-GM129325 and the Office of Cyber Infrastructure and Computational Biology, National Institute of Allergy and Infectious Diseases (Meng et al. 2023).

### Mass spectrometry analysis on TraF pulldown

Mass spectrometry analysis was performed as described in (Michaelis et al. 2024). The label-free quantification (LFQ) and the intensity-based absolute quantification (iBAQ) values were calculated using MaxQuant software (Cox and Mann 2008). An average of three independent measurements was taken. The error bars represent the standard deviation (SD).

## Results

### TraF is essential for pIP501 transfer

**Figure 1:**
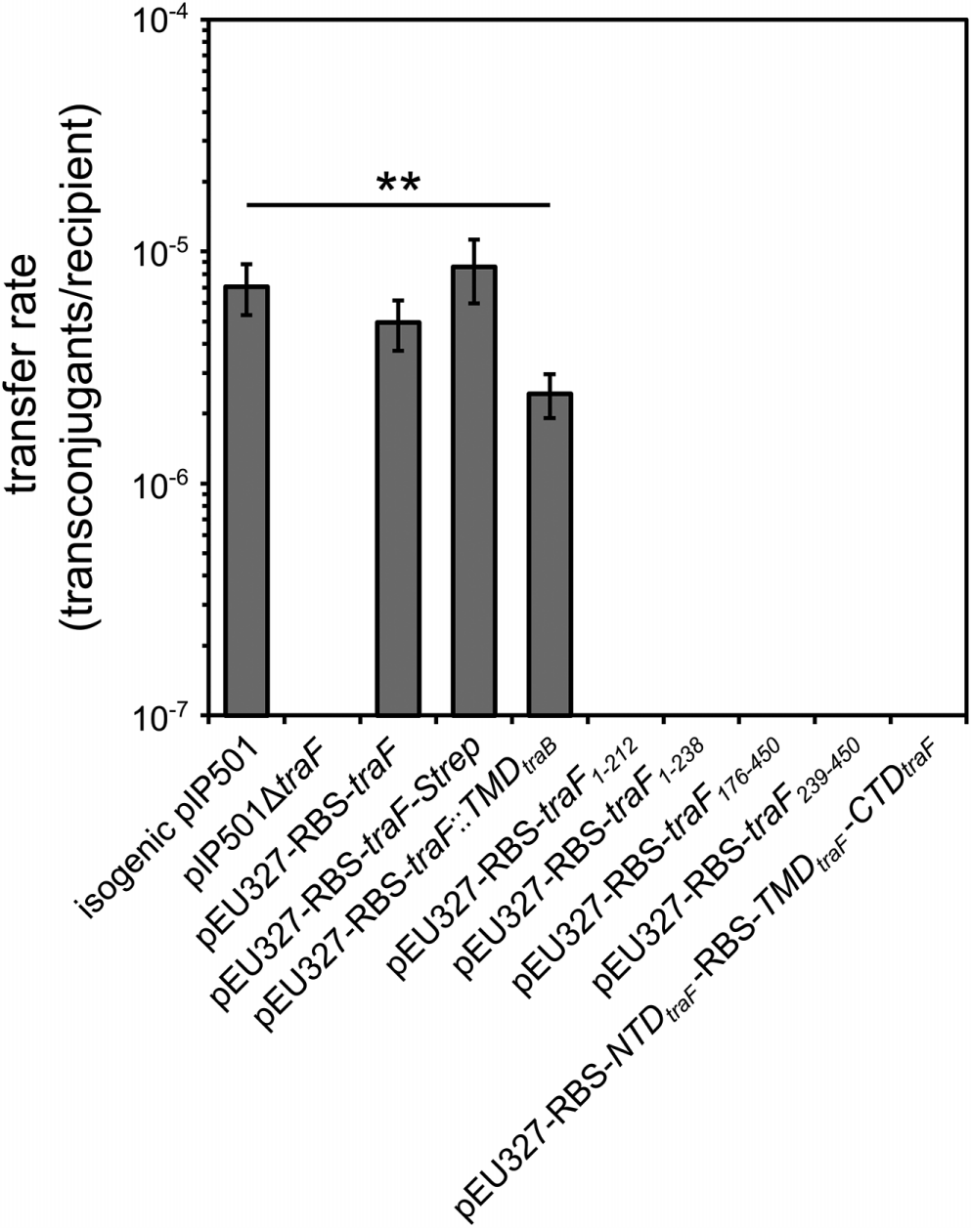
Biparental mating assays of isogenic pIP501, pIP501∆*traF*, and various pIP501∆*traF* complementation variants. *E. faecalis* JH2-2 (isogenic pIP501) and *E. faecalis* JH2-2 (pIP501∆*traF*, pEU327-RBS-*traF*) were used as reference strains. Complementation strains of the *traF* KO expressing the respective genes/gene variants in trans, *traF* (pEU327-RBS-*traF*), *traF* with C-terminal Strep-tag (pEU327-RBS-*traF*-Strep), *traF* with substituted transmembrane helix (TMD) against TMD of pIP501 transfer gene *traB* (pEU327-RBS-*traF:: TM*D*_traB_*). *E. faecalis* OG1X was used as the recipient. *n* = 3. Mean values are depicted with the standard error of the mean (s.e.m.). **P < 0.001 as determined by Mann–Whitney U test.

Deletion of *traF* resulted in a complete loss of pIP501 transfer between *E. faecalis* strains, while complementation with *traF in trans* (4.94 × 10^−6^ transconjugants/recipient) successfully restored the transfer rate to levels comparable to the isogenic pIP501 (7.06 × 10^−6^ transconjugants/recipient). Truncated TraF variants representing single domains (either N-terminal, N-terminal and transmembrane helix, transmembrane helix and C-terminal domain, or C-terminal domain alone), were unable to restore the transfer. Additionally, complementation with separately expressed *traF* domains inserted into pEU327 and transformed into *E. faecalis* JH2-2 (pIP501∆*traF*, pEU327-RBS-*NTD_traF_*-RBS-*TMD_traF_*-*CTD_traF_*), did not recover transfer. Substituting the transmembrane helix (TMD) of TraF with that of TraB (pEU327-RBS-*traF::TMD_traB_*) resulted in a transfer rate of 2.44 × 10^−6^, which showed a slight but statistically significant reduction compared to isogenic pIP501 (Fig. 1). The full numeric results are shown in Supplementary Table S3. The *in vivo* expression of *traF* variants was verified by immunoblotting (Supplementary Fig. S3). Exchanging the TMD_TraF_ with TMD_TraB_ of the same length did not affect the expression level of the protein. The expression level of the domains was lower than for the full-length proteins. This might result from partial degradation of the soluble protein domain (TraF-C). Bands of high molecular weight might indicate TraF aggregates.

### Structural analysis of soluble TraF domains (TraF-N and TraF-C)

**Figure 2:**
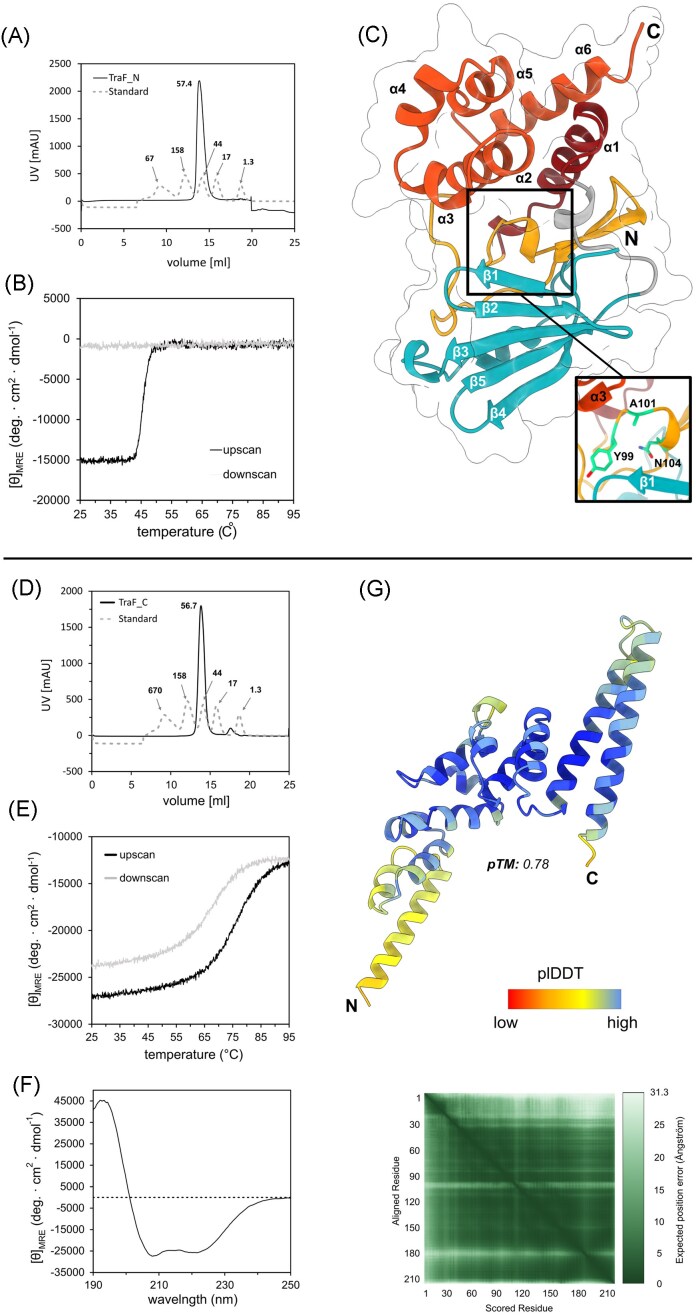
Biophysical characterization of TraF-N and TraF-C. (A) Gel filtration profile of TraF-N which eluted with an apparent molecular weight of 57.38 kDa appearing as a dimer in solution. (B) TraF-N CD temperature scan from 25°C to 95°C at 208 nm shows a melting temperature (Tm) of 47°C. (C) Crystal structure of the N-terminal domain of TraF (TraF-N) solved to 1.25 Å coloured by its distinct domains: C-terminal, α-helix rich domain (red), N-terminal, mainly β–sheet containing domain (blue), loop containing mutated amino acids of the kinase catalytic triad (orange). (D) Gel filtration profile of TraF-C which eluted with an apparent molecular weight of 56.7 kDa suggesting either dimer formation or an elongated structure. (E) CD temperature scan from 25°C to 95°C at 208 nm showing a melting temperature (Tm) of 72°C. (F) CD spectra of TraF-C showing a predominantly α–helical protein. (G) AlphaFold3 model of TraF-C coloured by plDDT values and respective predicted aligned error (PAE) matrix.

TraF-N was expressed and purified to high purity (Fig. S1). Crystallization attempts of His-TraF-N yielded crystals within one day. While the crystals from this construct diffracted to 3.2 Å only, optimization of the His-Tag-free TraF-N yielded crystals diffracting to 1.25 Å. Data collection and refinement statistics are shown in Supplementary Table S4. Residues 1–194 were visible in the electron density. Although gel filtration profiles suggest dimer formation under native conditions, TraF_1-212_ crystallized as a monomer. Circular dichroism revealed a melting temperature of ∼46°C without the ability to refold (Fig. 2 A, B).

The structure exhibits a pseudokinase (PK)-like fold with a distinct two domain structure: an N-terminal domain consisting of five β-sheets (aa 1–63) and a C-terminal domain consisting of five α-helices (aa 132–194). An additional α-helix (α1, aa 73–95) is connected to the C-terminal domain through a loop comprised of two antiparallel β-sheets as well as long stretches of unstructured regions (aa 96–131). β5 from the N-terminal domain is connected to α1 of the C-terminal domain through a linker harbouring a short α-helical patch (aa 64–72) containing the mutated catalytic triad (Fig. 2 C). A topological overview is shown in Supplementary Fig. S4 and a representative electron density map in Supplementary Fig. S5.

TraF_239-450_ (TraF-C) was purified to high purity using a combination of affinity chromatography and gel filtration (Supplementary Fig. S2). Unfortunately, TraF-C failed to crystallize. Like TraF-N, TraF-C eluted as a dimer during gel filtration (Fig. 2 D). CD spectra revealed that the protein is predominantly α-helical and highly stable with a melting temperature of 72°C which completely refolds upon cooling (Fig. 2 E, F). AlphaFold3 prediction (pTM: 0.78) shows a protein consisting of 11 α-helices of various lengths connected by short loops (Fig. 2 G).

### Search for structural homologs

**Figure 3:**
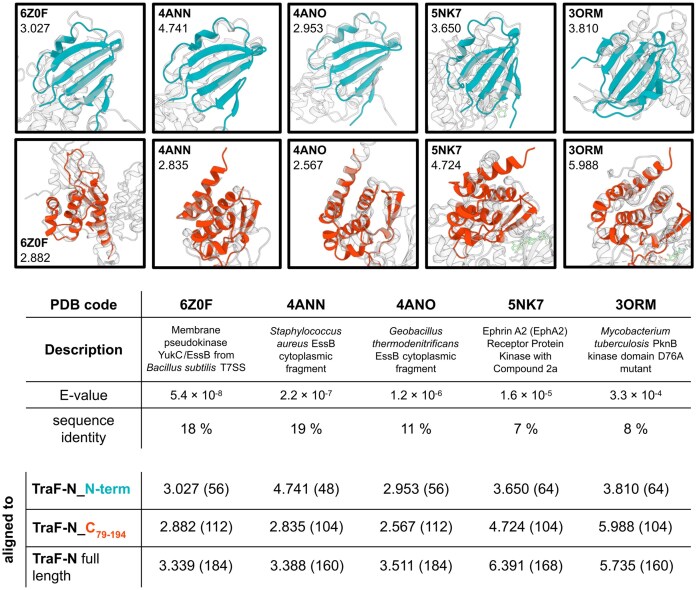
Cα-Cα alignment of the two distinct regions of TraF-N to crystal structures of structural homologs identified through FoldSeek. Alignments of TraF-N N-terminal region (aa 1-63): upper panels and TraF-N C-terminal region (aa 79-194): lower panels. PDB code and RMSD are depicted in the respective image as well as in the table. The number of aligned Cα-atoms is shown in brackets.

FoldSeek search (Van Kempen et al. 2024) of TraF-N revealed high similarity to the crystal structure of YukC from *Bacillus subtilis* (PDB: pdb_00006z0f) as well as the N-terminal cytoplasmic domains of EssB from *G. thermodenitrificans* (PDB: pdb_00004ano) and *S.s aureus* (PDB: pdb_00004ann). EssB (or YukC in *Bacillus* nomenclature) is a membrane protein which is essential for the secretion of ESAT-6 family proteins as well as recruitment of α-helical polymorphic toxins called LXG effectors, which inhibit the growth of bacterial competitors (Tassinari et al. 2022, Klein et al. 2024). Cα-alignment of TraF-N to YukC/EssB (pdb_00006z0f, pdb_00004ann, and pdb_00004ano) shows high structural similarity with an overall RMSD of 3.3 Å, 3.4 Å, and 3.5 Å, respectively, despite the low sequence identity of 18%, 19%, and 11%, respectively (Fig. 3). An alignment of full length YukC/EssB dimer to TraF-N is shown in Supplementary Fig. S6. Like EssB, TraF-N resembles a serine/threonine kinase fold containing a mutated catalytical triad (Y99, A101, N104) classifying both proteins as pseudokinases. Human Ephrin A2 (EphA2) Receptor Protein Kinase (PDB: pdb_00005nk7) was found to be the closest structural representative from mammalian source with an RMSD value of 6.4 Å. Restricting the results to eubacteria shows multiple structures of protein kinase B (PknB) from *Mycobacterium tuberculosis* (represented here by PDB: pdb_00003orm) amongst the top hits.

All found structures exhibit a similar two-domain structure with a mainly β-sheet N-terminal and a predominantly α-helical C-terminal part. Aligning only the respective regions of TraF-N to the structural homologs shows that the similarity of the N-terminal region is higher than that of the C-terminal region (Fig. 3).

In addition to the YukC C-terminal domain, Foldseek search for the AlphaFold model of TraF-C (aa 239–450) shows similarities with several structures of tetratricopeptide repeat (TPR) proteins. Both proteins feature a similar alternating arrangement of short α-helices with tetratricopeptide repeat protein (Fragment) from *Lentilactobacillus parafarraginis* F0439 (AF-G9ZPL5-F1-model_v6) and tetratricopeptide repeat protein from *Treponema phagedenis* (AF-A0AAE6IV90-F1-model_v6) with an RMSD of 7.16 and 7.71 respectively. A search for the full-length TraF AlphaFold model solely yields close matches to either the N- or the C-terminal domain.

### Deletion of *traF* does not influence the expression level of other Tra proteins

**Figure 4:**
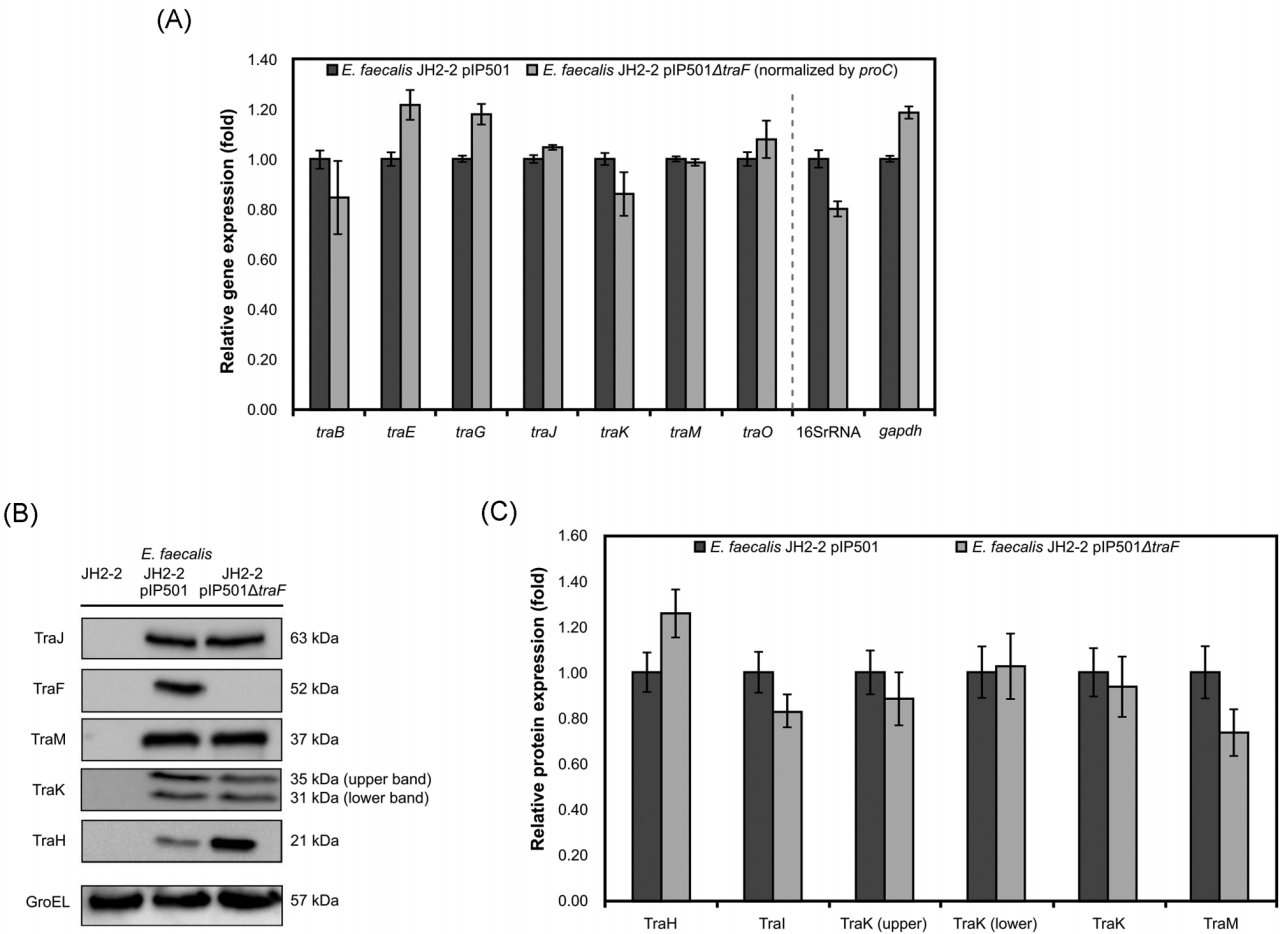
(A) Relative gene expression of the WT strain (*E. faecalis* JH2-2 pIP501) and the *traF* KO strain (*E. faecalis* JH2-2 pIP501*ΔtraF*) was compared by RT-qPCR. Gene expression was normalized to the expression of *proC*. 16S rRNA and *gapdh* genes were used as control. Means ± s.e.m, *n* = 3. (B) Protein levels of selected Tra proteins of *E. faecalis* JH2-2 pIP501 and the *traF* KO strain *E. faecalis* JH2-2 pIP501*ΔtraF* were compared by western blots. Plasmid-free *E. faecalis* JH2-2 was the negative control. GroEL was used as a loading control for quantification. (C) Protein expression of *E. faecalis* JH2-2 pIP501 and *E. faecalis* JH2-2 pIP501Δ*traF* were compared by quantitative western blots. Normalization was done with GroEL. Means ± s.e.m, *n* = 20 (TraH_B8_), *n* = 17 (TraJ_D4_), *n* = 14 (TraM_B8_), *n* = 12 (TraK). TraK = average of TraK upper and lower band.

As shown by quantitative RT-PCR, the transcription of selected *tra* genes, *traB, traE, traG, traJ, traK, traM*, and *traO* of the pIP501 *tra* operon was not significantly influenced by the *traF* KO (Fig. 4 A). The expression of the respective proteins is also not significantly impacted as shown by densitometric quantification of a western blot (Fig. 4B and C). Neither of the bands shows a significant difference in the *traF* KO strain. TraK has two start codons resulting in a lower band corresponding to 32.3 kDa and an upper band corresponding to 36.4 kDa.

### TraF interacts with TraM_B8_

**Figure 5:**
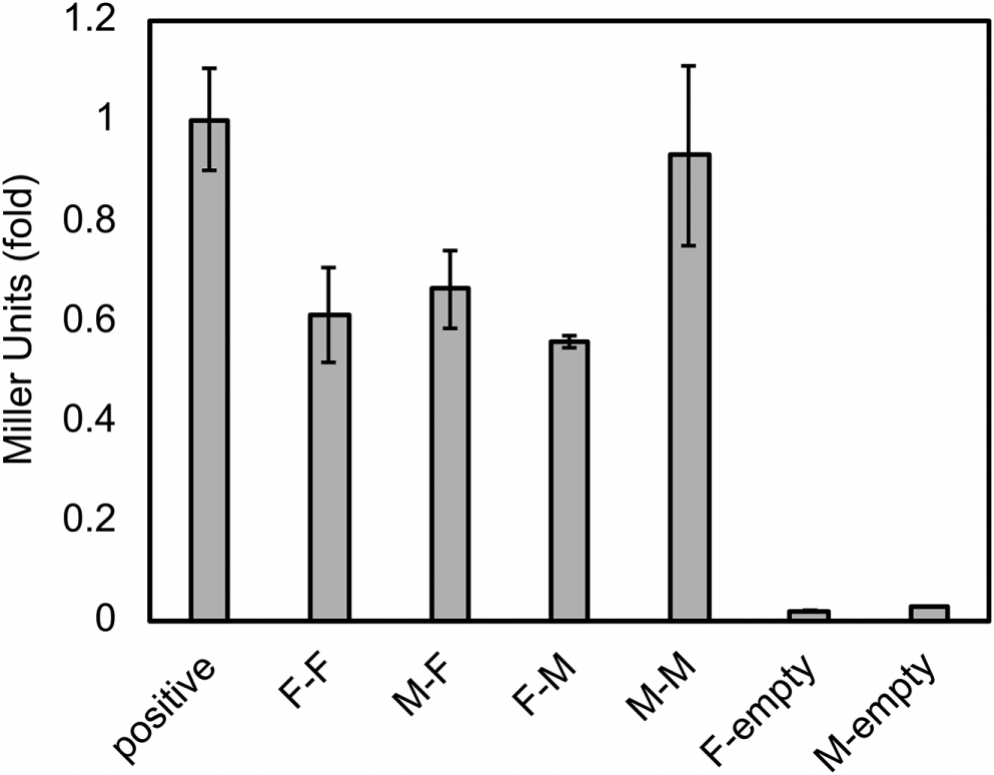
B2H assay showing interactions between TraF and TraM_B8_. As positive control, the GCN4 leucine zipper was fused to the T18 and T25 fragments. The negative control is the respective empty vector as described in Kohler et al. (2017). Means ± standard deviation, *n* = 6 except for TraM_B8_-TraF (*n* = 4).

The B2H system was applied to study TraF interactions with other pIP501 Tra proteins. The only interaction of TraF was found with TraM_B8_, and with itself. β-galactosidase assays of TraM_B8_ and TraF fused either as prey or bait to T18 and T25 fragments of adenylate cyclase showed comparable interaction strength as the dimeric interaction of TraF with itself. In comparison, the TraM_B8_ interaction with itself, as shown in Kohler et al. (2017), was stronger than the TraF-TraF interaction and nearly as strong as the positive control (Fig. 5). B2H interaction for TraM_B8_ with TraG_B1_, TraJ_D4_, and TraL_B6_ are shown in Supplementary Fig. S7.

### Full-length TraF shows structural similarity to YukC

Due to the lack of suitable crystals, AlphaFold was applied to predict homo-multimer models of full-length TraF. Due to the presence of a long transmembrane domain, predictions of a single protein copy result in a “collapsed” formation where the N-terminal domain folds onto the C-terminal domain due to a lack of hydrophobic environment for the transmembrane stretch. By utilizing AlphaFold3, we imitated a lipid bilayer by including parameterized phosphatidyl glycerol (PG) and cardiolipin (CDL) molecules in the prediction.

**Figure 6:**
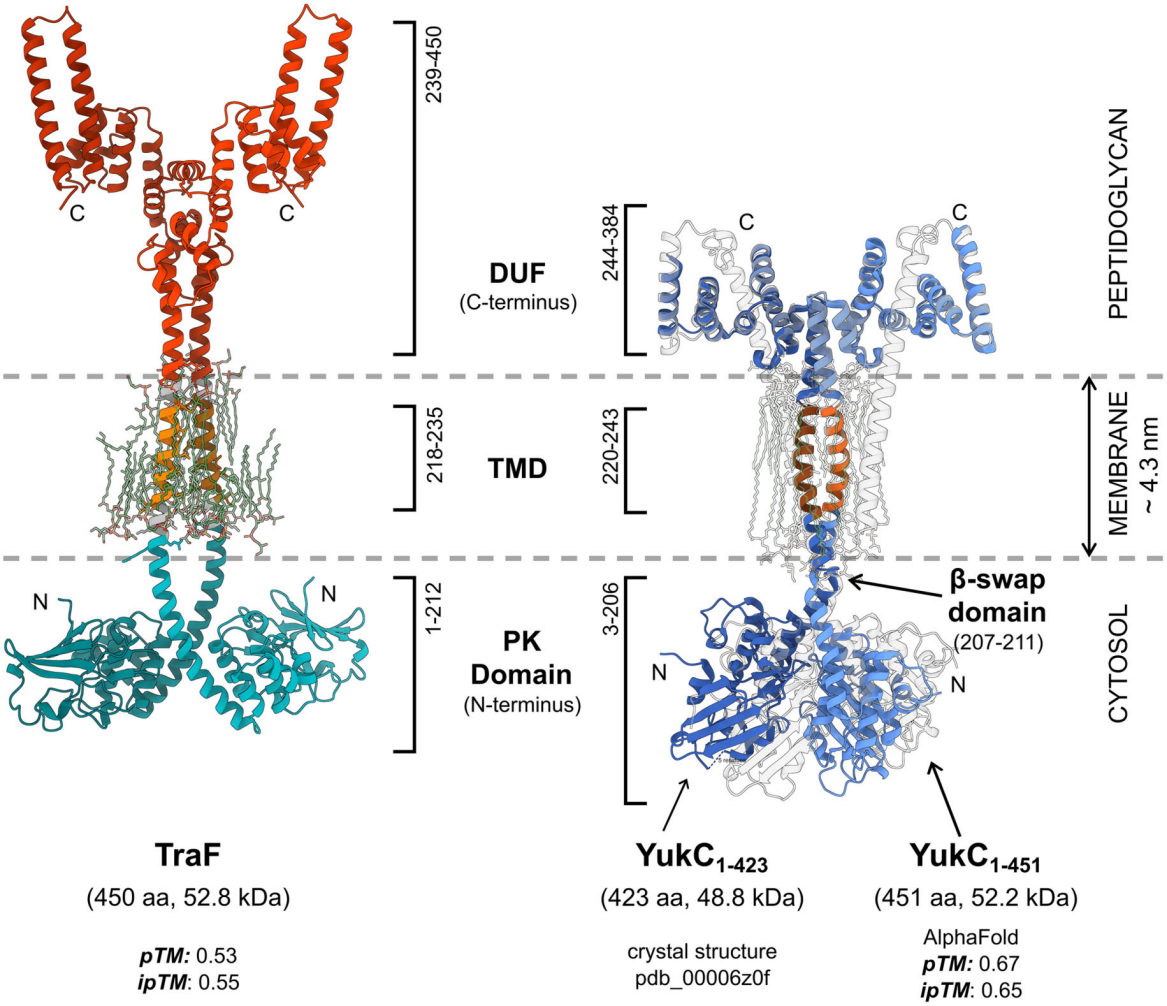
Comparison of full-length TraF and YukC (pdb_0006z0f), a transmembrane protein from *B. subtilis* T7SSb. AlphaFold model of a TraF homodimer with 15 PG and 5 CDL molecules as membrane mimetics coloured by distinctive domains showing the N-terminal PK-domain (blue), the membrane-embedded TMD (orange), and the α-helical C-terminal extracellular domain (red). The same arrangement is found within YukC crystal structure: dark blue; AlphaFold model with 20 PG: white.

Different oligomeric assemblies of TraF were tested (dimer and trimer). All predictions with the addition of membrane mimetics show a symmetric arrangement of the protein chains around a vertical central axis (Supplementary Fig. S8). The TMH domain, as identified by DeepTMHMM is surrounded by a random distribution of added PGs and CDLs forming a lipid bilayer (Fig. 6). The dimeric assembly showed the overall best pTM and ipTM scores (0.53 and 0.55, respectively) and a remarkable similarity to the crystal structure of the YukC dimer from *B. subtilis* T7SSb (pdb_00006z0f).

The TMD predicted by the DeepTMHMM server correlates with the TMD as displayed by the AlphaFold3 prediction. Both proteins feature a cytosolic PK-domain and an all-α-helical C-terminal domain which likely reaches into the peptidoglycan layer. TraF prediction lacks the characteristic β-swap domain present in YukC located at the border of the cytosolic membrane leaflet. However, the prediction scores remain too low to draw any conclusions on residues involved in intermolecular dimer interactions to reliably compare them to interactions observed for YukC.

The highest structural similarity to full length TraF was found to the two-domain transmembrane pseudokinase YukC from *B. subtilis* T7SSb which crystallized as a dimer. The PK-dimer of YukC interacts with the FHA-domain of YukB (Oka et al. 2025). Aligning the crystal structure of the FHA-domain from EssC (pdb_00005fwh) as well as the AlphaFold model of YukB-FHA domains (UniProtID: C0SPA7, domain 1: aa 1–91 and domain 2: aa 95–207) to AlphaFold predictions of all pIP501 Tra proteins showed that none of the pIP501 Tra proteins adopt an FHA-domain fold. Additionally, we investigated if a YukB (EssC) homolog might be present in our system by aligning the AlphaFold model of YukB (pTM: 0.66) to all Tra proteins. Results showed expected similarities to the ATPase subunits of TraE_B4_ and TraJ_D4_ but revealed no further structural homologies (data not shown).

### Mass spectrometry of the TraF pulldown reveals potential interaction partners

**Figure 7:**
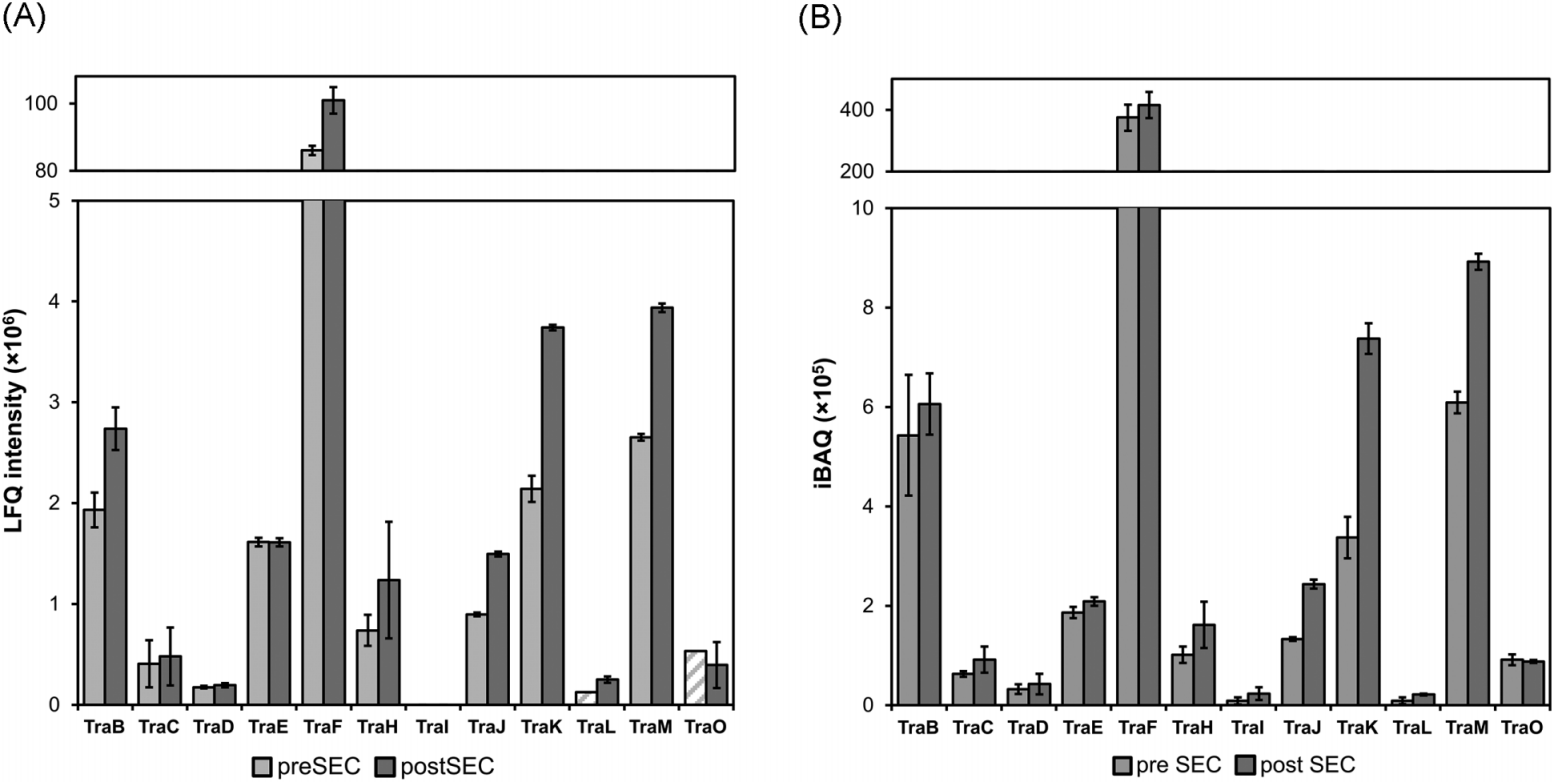
Mass spectrometry analysis of a TraF pull-down experiment. (A) LFQ and (B) iBAQ intensities for each Tra protein identified in the TraF pulldown. Average of three runs, error bars: standard deviation. For identified TraL_B6_ and TraO proteins, LFQ intensities derived from MS1 peptide signal intensities were obtained in only one of three runs, preventing statistical analysis and error bar calculation (hatched).

To identify potential interaction partners of TraF, mass spectrometry analysis of the TraF-Strep pulldown fraction was performed. TraF-Strep as well as TraB-TraO were expressed in *P. megaterium* and purified using a Strep affinity column. Part of the sample was subjected to gel filtration prior to MS analysis (post SEC). The iBAQ was calculated to enable the comparison of the relative protein abundance of different Tra proteins within one sample. LFQ values are presented to show consistency between various measurements (Fig. 7).

TimsToF data revealed that all Tra proteins apart from TraG_B1_ (the lytic transglycosylase) and TraN (the transcriptional repressor) were present after the pulldown. Additionally, only low amounts of TraJ_D4_ (coupling protein), TraI (putative membrane anchor of TraJ_D4_), and TraO (putative adhesin) were detected. Gel filtration after the pulldown had almost no effect on the amount of detected Tra proteins apart from TraL_B6_, a protein likely involved in T4SS complex formation, which got enriched. The raw LFQ and iBAQ values are shown in Supplementary Table S5.

## Discussion

In this study, we investigated TraF, an essential T4SS transmembrane protein from the *E. faecalis* conjugative plasmid pIP501. We show the 1.25 Å crystal structure of the N-terminal domain which exhibits a pseudokinase fold. A structural homology search revealed various pseudokinases including YukC, a T7SSb protein with the highest resemblance to TraF. Structure predictions of full length TraF suggest a distinct, membrane spanning, two-domain structure like YukC. TraF-pull down analysis showed that 12 out of the 14 Tra proteins tested were isolated together with TraF. B2H assay revealed the strongest interaction of TraF with TraM_B8_. TraF was demonstrated to be an essential transfer protein, where the full-length protein was required for conjugative pIP501 transfer among *E. faecalis* cells.

Each of the soluble TraF domains alone as well as co-expression of the separated domains with TMD_TraF_ could not restore the transfer. The replacement of the TMD_TraF_ in the full-length TraF by the sequentially unrelated TMD of the same length (TMD_TraB_) complemented the *traF*-KO with a slight but statistically significant reduction of the transfer rate (∼0.5 log). Therefore, the correct spatial organization of both TraF domains but not necessarily the sequence of the TMD appears to be crucial for transfer.

**Figure 8:**
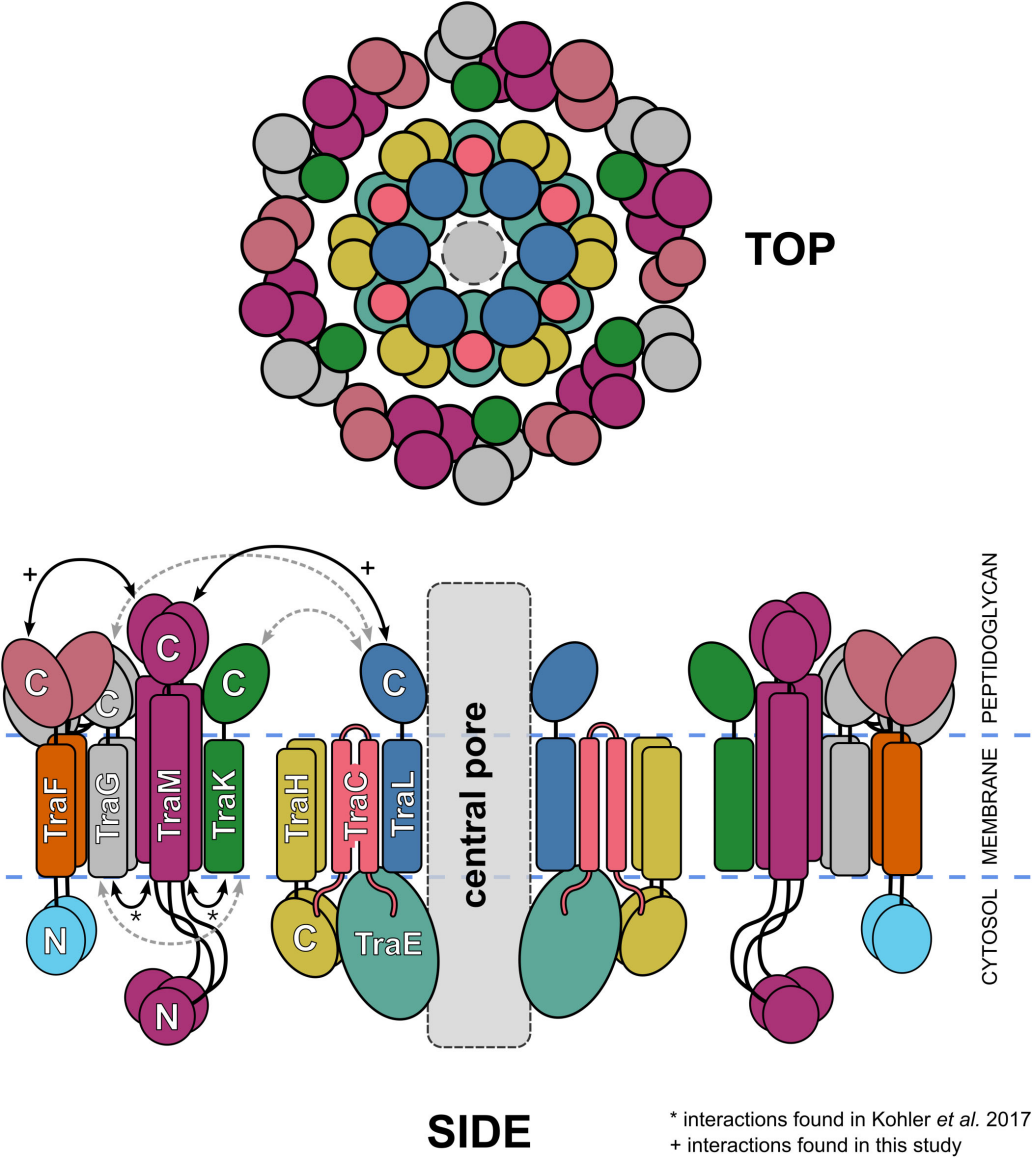
Side and top view of the postulated arrangement of the *E. faecalis* T4SS MPF complex components TraL_B6_ (dark blue), TraC_B3_ (salmon), TraH_B8_ (khaki), TraK (green), TraG_B1_ (grey), TraM_B8_ (violet), TraE_B4_ (teal), and TraF (N-terminal domain: cyan, transmembrane domain: orange, C-terminal domain: pale red). The domain arrangements as well as interactions are based on experimental data from this study (marked with +), B2H assays (Kohler et al. 2017, marked with *) as well as AlphaFold predictions (Breidenstein et al. 2025). For clarity reasons TraB and TraI were not included in this scheme. Dashed grey lines indicate putative interactions based on preliminary data.

TraF shows a strong interaction with TraM_B8_, a crucial building block of the MPF complex. TraM_B8_ strongly interacts with TraL_B6_, the coupling protein TraJ_D4_ and to a weaker extent with the lytic transglycosylase TraG_B1_ (Kohler et al. 2017) suggesting its crucial role in the formation of the core complex. TraL_B6_ is believed to form the central component of the MPF complex in analogy to solved structures of G- T4SSs (Paillard et al. 2025, Waksman 2025) as well as the model structure of the monoderm pCF10 system (Breidenstein et al. 2025). As TraM_B8_ interacts with both TraL_B6_ and TraF but there is no evidence on direct interaction of TraF with TraL_B6_, we propose that TraM_B8_ functions as an intermediate component required for the complex formation (Fig. 8).

No TraF homologs (both sequence-based or structural) have been found in other T4SSs. Search for TraF structural homologs mainly revealed kinases and pseudokinases from bacterial as well as mammalian origin. Aligning the sub-domains of TraF-N (TraF-N_N and TraF-N_C, respectively) to the identified homologs (YukC, EssB, EphA2 Receptor Protein Kinase, and PknB kinase) showed a high similarity to their respective counterparts. However, the RMSD for the full-length protein remains rather low due to a different orientation of the sub-domains relative to each other. For some of the structures, this is likely caused by the presence of a ligand bound in the active site.

Pseudokinases work as scaffolds, adapters, allosteric regulators, and signal transducers. This function is partially mediated by their ability to convey conformational changes upon substrate binding (Zeqiraj and Van Aalten 2010, O’Boyle et al. 2025). Recent reports suggest that the pseudokinase domain (exemplary stated on YukC) may be involved in coupling conformational changes between T7SS proteins due to its interaction with the YukB forkhead-associated (FHA) domain (Tassinari et al. 2022).

*In vitro*, the YukC PK-domain was shown to be unable to bind ATP presumably due to a bulky F26 residue pointing inside the putative ATP binding cleft (Tassinari et al. 2022). TraF-N has a valine V20 at the respective position which might allow ATP-binding. However, TraF-N only shows two small cavities at the putative ATP binding site which are separated by Y60 and Y117 residues making substrate binding very unlikely (Supplementary Fig. S9). Like YukC, TraF features a mutated catalytic triad reinforcing the inability of potential phosphate transfer.

The two-domain transmembrane pseudokinase YukC from *B. subtilis* T7SSb which crystallized as a dimer has the highest structural similarity to full length TraF. The PK-dimer of YukC interacts with the FHA-domain of YukB (Oka et al. 2025). Additionally, YukC showed interactions with all *B. subtilis* T7SSb operon proteins. Therefore, it likely acts as an interaction hub for the assembly of the system (Tassinari et al. 2022). YukB adopts a multi-domain fold with two FHA-domains at the N-terminus, a two-fold transmembrane domain as well as three ATPase domains (DI, DII, DIII) and a domain of unknown function (DUF) (Rosenberg et al. 2015). Only the pIP501 ATPase subunits of TraE_B4_ and TraJ_D4_ showed structural similarity to YukB domains. This likely indicates that the ancestor of a membrane-bound pseudokinase (*traF*) might have been the only T7SS gene that has been horizontally acquired by a T4SS lacking its original interaction partner.

Interestingly, conjugative plasmid pLS20 from *B. subtilis* features a *virB11* homolog which is absent from other monoderm T4SSs (Bauer et al. 2011, Val-Calvo et al. 2021, Breidenstein et al. 2025). Since pIP501 is a broad-host-range conjugative plasmid, it can transfer to various bacterial genera containing T7SSs (e.g. staphylococci and bacilli). As pIP501 is not stably maintained in *B. subtilis*, it might have integrated into the chromosome and acquired the *traF/essB* gene through recombination events.

YukC features multiple proline residues within the transmembrane stalk which are postulated to be important for signal sensing and transduction. The TraF stalk domain lacks prolines ruling out a similar signal transduction pathway as in the T7SS counterpart.

In several mycobacterial conjugative plasmids, a combination of T4SS VirB4, VirD4, VirB8 (TcpC_pCW3_-like protein) and relaxase homologs were found together with a T7SSa. Both systems were essential for conjugation (Ummels et al. 2014). Plasmid-encoded mycobacterial T4SSs and T7SSs likely evolutionarily co-diverged which may explain the presence of a YukC-like protein fold in both systems (Mortimer et al. 2017).

The cryo-EM structure of the T7SSb core complex consisting of YukC, the ATPase YukB and the small ubiquitin-like protein YukD shows direct interaction of the YukC PK-domain with the FHA domain of YukB (Oka et al. 2025). A model consisting of six YukC dimers was generated and predicted to sit on top of the YukB FHA domain in a symmetric assembly forming a hexameric ring-like structure. A pentameric assembly for the central VirB6-like proteins was observed in the T4SS_R388_ structure and similarly predicted in the T4SS_pCF10_ (Macé et al. 2022, Breidenstein et al. 2025). AlphaFold predictions of TraL_B6_ showed that both a pentameric and a hexameric assembly are possible. In case of T7SSb, YukC dimers interact with YukB but do not interact with themselves in the PK-domain. TraF shows a strong interaction with TraM_B8_, which in turn forms an interaction hub for other MPF proteins where TraF might act as a stabilization factor.

TPR proteins mediating protein-protein interactions are also structurally homologous to TraF-C (Perez-Riba and Itzhaki 2019). Therefore, TraF-C might be crucial for the strong interaction observed with TraM_B8_. For the AlphaFold model, the addition of membrane mimetics was necessary to improve the structure prediction as compared to the dimer without lipids.

Comparing the predicted TraF dimer with the crystal structure of YukC revealed a similar domain arrangement. The YukC homodimer features an additional β-swap domain located within the TMH part close to the PK-fold domain which is not present in the TraF dimer prediction. The YukC TMH domain harbors a proline which introduces a kink into the α-helix structure allowing a more intimate contact between the transmembrane helices. A mutation of a proline to an alanine at this position resulted in decreased T7SS killing activity (Tassinari et al. 2022). TraF contains no proline within the TMH and therefore exhibits a straight arrangement of the transmembrane helices instead.

A recent publication on additional T7SSs types revealed further TraF-like proteins. TsxD was identified as a kinase where x stands for the different subsystems: c, d, e, and j (Garrett et al. 2024). Aligning a TraF dimer to the AlphaFold models of TscD, TsdD, TseD, and TsjD with membrane mimetics shows a very similar bitopic arrangement for all kinases except for TscD from *Paenibacillus azoreducens* (Supplementary Fig. S10).

Mass spectrometry revealed that the lytic transglycosylase TraG_B1_ and the conjugative repressor TraN are not present in the TraF pulldown. The absence of TraG_B1_ is likely due to its weak interaction with other Tra proteins, as exemplary shown in a B2H assay with TraM_B8_ (Kohler et al. 2017). Additionally, TraG_B1_is a peptidoglycan degrading enzyme. It is postulated to be assembled on the periphery of the MPF complex and thus more likely removed from the complex during purification. The absence of TraN is expected as it is a cytosolic DNA-binding protein very unlikely forming part of the core complex (Kohler et al. 2018). The putative adhesin TraO and the TraJ_D4_ membrane anchor TraI were detected but not reproducibly quantifiable due to their low occurrence. We assume that TraO is not part of the core complex but plays a role in mediating cell-to-cell contact. TraI likely functions as a membrane anchor for the coupling protein TraJ_D4_ abundantly found in the pulldown. As shown by B2H assays, TraJ_D4_ interacts with the core complex, therefore direct involvement of the membrane anchor TraI in the complex is not required. TraL_B6_, the putative core of the MPF complex, got enriched instead. This is likely due to the increase of the TraL_B6_ signal in the post-SEC sample as unspecifically bound proteins were removed.

In summary, TraF is the first T4SS component that shares extensive structural homology to a T7SS pseudokinase. TraF plays an important role in the assembly of the secretion system through interactions with the transmembrane protein TraM_B8_. Due to structural similarities to T7SS, there might be more common features between these systems and conjugative T4SS than expected. TraF interactions with other Tra proteins provided insight into the putative composition of the T4SS_pIP501_ core complex. The lack of a TraF-like protein in other T4SS (both mono- and diderm) points towards horizontal acquisition of this gene from other secretion systems.

## Supplementary Material

uqag009_Supplemental_File

## Data Availability

The original contributions presented in the study are included in the article/supplementary material; further inquiries can be directed to the corresponding authors.
