# Supplementary material for: Crucial Gram-positive type IV secretion system protein TraF is a structural homolog of type VII secretion system protein EssB/YukC
Source: Microlife. 2026 Mar 23;7:uqag009. doi: 10.1093/femsml/uqag009 (PMC13037476; doi:10.1093/femsml/uqag009)
Supplement: uqag009_Supplemental_File [file uqag009_supplemental_file.pdf]

Supplementary material for manuscript "Crucial Gram-positive type IV secretion system protein TraF is a structural homolog of Type VII secretion system protein EssB/YukC"

Supplementary Table S1 Bacterial strains and plasmids used in this work. Amp^{r}, ampicillin resistance; Cm^{r}, chloramphenicol resistance; Em^{r}, erythromycin resistance; Fus^{r}, fusidic acid resistance; Gent^{r}, gentamicin resistance; Km^{r}, kanamycin resistance; MLS^{r}, macrolide-lincosamide-streptogramin B resistance; Sm^{r}, streptomycin resistance; Spec^{r}, spectinomycin resistance; *tra*$^{+}$, transfer proficient.

**Strain or plasmid**	**Genotype or description**	**Reference or source**
**Strain**		
E. coli DH5	F^{-} φ80*lac*ZΔM15 Δ(*lac*ZYA-*arg*F)U169 *rec*A1 *end*A1 *hsd*R17(r$_{K}^{-}$, m$_{K}^{+}$) *pho*A *sup*E44 λ^{-}*thi*-1 *gyr*A96 *rel*A1	Invitrogen
E. coli EC1000	F^{-} Δ(*araA–leu*)^{7697}, [*araD139*] *B/r*, Δ(*codB–lacI*)^{3}, *galK16*, *galE15* (GalS), λ^{-}, *e14*$^{-}$, *relA1*, *rpsL150* (Sm^{r}), *spoT1*, *mcrB1*	(Leenhouts *et al.* 1996)
E. coli BTH101	*lacZ*$^{+}$ F^{-}; *cya*-99*, ara*D139*, gal*E15*, gal*K16*, rps*L1 (Sm^{r})*, hsd*R2*, mcr*A1*, mcr*B1	Euromedex
E. coli TOP10	F^{-}*mcr*A Δ(*mrr-hsd*RMS-*mcr*BC) φ80*lac*ZΔM15 Δ*lac*X74 *rec*A1 *ara*D139 Δ(*ara-leu*)7697 *gal*U *gal*K λ–*rps*L(Sm^{r}) *end*A1 *nup*G	ThermoFisher Scientific
E. faecalis JH2-2	Derivative of *E. faecalis* JH2, Rif^{r}, Fus^{r}	(Jacob and Hobbs 1974)
E. faecalis OG1X	Protease-negative mutant of *E. faecalis* OG1-10, Sm^{r}	(Ike *et al.* 1983)
**Plasmid**		
pIP501	*tra*$^{+}$, Cm^{r}, MLS^{r}	Berliner Hochschule für Technik
pUC18	Amp^{r}	ThermoFisher
pUC18-UPS-*traF*	pUC18 with *traF* upstream regions at BamHI/EcoRI sites	This study
pUC18-UPS-DWS-*traF*	pUC18-UPS-*traF* with *traF* downstream regions at BamHI/EcoRI sites	This study
pKA	pCJK47 *aacA-aphD* at BglII site; Em^{r} Gent^{r}	(Arends *et al.* 2013)

pKAΔ*traF*	pKA with *traF* up- and downstream regions at PstI/EcoRI sites	This study
pIP501Δ*traF*	pIP501 *traF* in-frame deletion	This study
pEU327	*E. coli* / G+ bacteria shuttle plasmid, $Spec^r$ *xylA* promoter	(Eichenbaum *et al.* 1998)
pEU327-RBS-*traF*	pEU327 with RBS-*traF*	This study
pEU327-RBS-Strep	pEU327 with RBS-Strep	This study
pQTEV	P_{t4} $lacI^q$ His_7 Amp^r	(Scheich *et al.* 2004)
pUT18-X*	Derivative of high-copy number plasmid pUC19, aa 225–399 of adenylate cyclase (CyaA) (T18), multiple cloning site (MCS) at 5´end of T18. Domain of prey* (X) fused to T18 fragment, Amp^r	(Karimova *et al.* 1998)
pUT18C-X*	Derivative of high-copy number plasmid pUC19, aa 225–399 of CyaA (T18), MCS at 3′ end of T18. Domain of prey* (X) fused to T18 fragment, Amp^r	(Karimova *et al.* 1998)
pKT25-Y*	Derivative of low-copy number plasmid pSU40, first 224 aa of CyaA (T25), MCS at 3′ end of T25. Domain of bait* (Y) fused to T25 fragment, Km^r	(Karimova *et al.* 1998)
pKNT25-Y*	Derivative of low-copy number plasmid pSU40, first 224 aa of CyaA (T25), MCS at 5′ end of T25. Domain of bait* (Y) fused to T25 fragment, Km^r	(Karimova *et al.* 1998)
		* X=prey, Y=bait

Supplementary Table S2 Oligonucleotides used in this work.

Name	Sequence (5‘- 3‘)	Nucleotide position/reference
***traF* in-frame deletion**		
PstI_ups for orf6 ko fw	CGG**CTGCAG**AACTCAACAGAATTAAGGCA	5613-5632[a]
XbaI_ups for orf6 ko rev	GGC**TCTAGA**GTATTTCATTTTTTTCTCCCT	6757-6777[a]
BamHI_dws for orf6 ko fw	CAG**GGATCC**GCTGAAAAAGATTAGGAAGG	8107-8126[a]
C_EcoRI_dws for orf6 ko rev	CAG**GAATTC**CATTCGCCACCATTGCC	889-905[b]
**pKA sequencing**		
pKA fw	GGAGACTACTTATTATGTAA	(Kohler *et al.* 2018)
pKA rev	GCGCTTGTAATGTCATAT	(Kohler *et al.* 2018)
**Screening in-frame deletion**		
A Screen orf6 Mutant fw	CCACTTACTTAACGTCAG	5566-5583[a]
B Screen orf6 Mutant rev	CAAGAAGCGTACCGTCT	1041-1057[b]
C Screen orf6 Mutant fw	GCAATACGCTAGATGTTG	5755-5772[a]
D Screen orf6 Mutant rev	CCACCGCCATAATTATAC	471-488[b]
***traF* complementation**		
pEU327_BstYI_traF fw	CGC**GGATCT**CGAAAATTCAATAGAAGGG	6742-6760[a]
pEU327_SalI_traF rev	GGC**GTCGAC**AATCACCAACCTTCCTAAT	8117-8135[a]
pEU327_SalI_traF1-212 rev	GAG**GTCGAC**TTATTATCTGCGAATTTTTCGGAC	7387-7404[a]
pEU327_SalI_traF 1-238 rev	GCG**GTCGAC**TTATTAATTTACTTTCATAAGTAAA	7464-7482[a]
pEU327_BstYI_traF239-450F	CAC**GGATCT**TTAGAAGGGAGTATTAT**ATG**GAGAAGAAACAAGTTACG	7483-7500[a]
Mut_traF176-450 fw	CATTTTTTTCTCCCTTCTATTG	6750-6771[a]
Mut_traF176-450 rev	AGCTTGGAATCATTAAGAG	7294-7312[a]
Ins_212-traF rev	ctgcactaggagcagcaagaagggagaaaaaaatgAATAAAAGCTTAAAAATTGCG	7405-7425[a]
Ins_212 traF fw	aagatcctagtgctgctcctgctgcagactactaTCTGCGAATTTTTCGGAC	7387-7404[a]
Mut_traF_TMD1-B fw	aacaattaaggctgttaaaacaccttttatTTTATTTCTGCGAATTTTTCG	7390-7410[a]
Mut_traF_TMD1-B rev	GtagttggtgggattgcttgtgcgaagattATGAAAGTAAATGAGAAGAAAC	7471-7492[a]
GA_RBS-traF fw	gtggctccaagcgctCGAAAATTCAATAGAAGGGAG	6974-7009[a]
GA_RBS-traF rev	tgaatgccggatcgatATCTTTTTCAGCGTTTGTTAATTC	5602-5641[a]
GA_traF Strep fw	TGCGGGTGGCTCCAAGCGCTATCTTTTTCAGCGTTTGTTAATTC	8095-8118[a]
GA_traF Strep rev	TATTTGAATGCCGGATCGATCGAAAATTCAATAGAAGGGAG	6739-6762[a]

GA_traF$_{1-212}$ Strep fw	tgcgggtggctccaagcgctTCTGCGAATTTTTCGGAC	7387-7404[a]
GA_traF$_{1-212}$ Strep rev	tatttgaatgccggatcgatCGAAAATTCAATAGAAGGGAG	6742-6762[a]
GA_traF$_{1-238}$ Strep fw	tgcgggtggctccaagcgctATTTACTTTCATAAGTAAAACCG	7460-7482[a]
GA_traF$_{1-238}$ Strep rev	tatttgaatgccggatcgatTCGAAAATTCAATAGAAGGG	6741-6760[a]
GA_pEU327_C Strep fw	ATCGATCCGGCATTCAAATACAGATGCATTTTATTTC	(Berger *et al.* 2022)
GA_pEU327_C Strep rev	AGCGCTTGGAGCCACCCG	(Berger *et al.* 2022)
**pEU327 sequencing**		
pEU327 fw	CTTGCCAGTCACGTTACG	(Eichenbaum *et al.* 1998)
pEU327 rev	GATCAGCGATATCCACTTC	(Eichenbaum *et al.* 1998)
**pRBBm59 cloning**		
TraJ_pCStrep1623 fw	atat**AGATCT**ATGACTAGTTTATTAGCAGAATCAG	2145-2169[b]
TraJ_pCStrep1623 rev	aatt**GCGGCCGC**AAAAAATGGTAATTCG	3785-3798[b]
fw_TraJ_pRBBm59	atat**GCTAAGC**AATAAAAAGGGGAAA**GGATCC**ATGACTAGTTTATTAGCAGAATC	2145-2167[b]
rev_TraJ_pRBBm59	aatt**GAGCTC**TTATTTCTCGAACTGCG	3651-3657[b]
fw_TraF_pRBBm59	atat**GGATCC**ATGAAATACAAAATCTTGAA	6769-6788[a]
rev_TraF_pRBBm59	aatt**GCGGCCGC**AAATCTTTTTCAGCGTTTG	8102-8117[a]
**pRBBm59 sequencing**		
Screen_pRBBm59 fw	CGCAACGTCTGGAAATCGTG	(Berger *et al.* 2022)
Screen_pRBBm59 rev	GCGCATTCACAGTTCTCCGC	(Berger *et al.* 2022)
**bacterial-two-hybrid plasmids cloning**		
N-terminal fw	atat**CTGCA**aATGAAATACAAAATCTTGAAAAAT	6769-6792[a]
N-terminal rev	aatte**GGATCC**aaTAATTCAGCAGAAAAGAAATTC	7347-7368[a]
membrane fw	atat**CTGCA**aCAGGACAAGAAAGCAG	73669-738[a]
membrane rev	aatt**GGATCC**aaCTCATTTACTTTCATAAGTAAAAC	7462-7485[a]
C-terminal fw	atat**CTGCA**aAAGAAACAAGTTACGGC	7486-7502[a]
C-terminal rev	aatt**GGATCC**aaATCTTTTTCAGCGTTTGT	8101-8118[a]
**pQTEV cloning**		
TraF-N fw (BamHI)	gcccg**GGATCC**ATGAAATACAAAATCTTG	6769-6786[a]
TraF-N rev (KpnI)	ttt**GGTACC**CTATCTGCGAATTTTTCG	7390-7404[a]
TraF-C fw (BamHI)	attg**GGATCC**GAGAAGAAACAAGTTACG	7483-7500[a]
TraF-C rev (KpnI)	atcc**GGTACC**CTAATCTTTTTCAGCG	8106-8121[a]

restriction sites are shown in **bold**, ribosomal binding sites are underlined, and 5' overhangs are indicated by using small letters.
GenBank accession numbers for pIP501 sequences used in this study: [a] L39769.1 and [b] AJ505823.1.
All primers without reference in the table were designed in this study.

Supplementary Table S3 Transfer rates obtained in biparental mating assays.

**Matings: Testing of various TraF domains**		
**donor**	**recipient**	**transfer rate (transconjugants/recipient)**
E. faecalis JH2-2 (pIP501)	*E. faecalis* OG1X	$7.06 \times 10^{-6} \pm 1.75 \times 10^{-5}$
E. faecalis JH2-2 (pIP501Δ*traF*)		below detection limit
E. faecalis JH2-2 (pIP501Δ*traF*, pEU327-RBS-*traF*)		$4.94 \times 10^{-6} \pm 1.2 \times 10^{-5}$
E. faecalis JH2-2 (pIP501Δ*traF*, pEU327-RBS-*traF*-Strep)		$8.61 \times 10^{-6} \pm 2.62 \times 10^{-5}$
E. faecalis JH2-2 (pIP501Δ*traF,* pEU327-RBS-*traF*::TMD_{traB}		$2.44 \times 10^{-6} \pm 5.23 \times 10^{-6}$
E. faecalis JH2-2 (pIP501Δ*traF*, pEU327-RBS-$traF_{1-212}$)		below detection limit
E. faecalis JH2-2 (pIP501Δ*traF*, pEU327-RBS-$traF_{1-238}$)		below detection limit
E. faecalis JH2-2 (pIP501Δ*traF*, pEU327-RBS-$traF_{239-450}$)		below detection limit
E. faecalis JH2-2 (pIP501Δ*traF*, pEU327-RBS-$traF_{176-450}$)		below detection limit
E. faecalis JH2-2 (pIP501Δ*traF*, pEU327-RBS-NTD_{traF}-RBS-TMH_{traF}-CTD_{traF})		below detection limit

mean values are given with standard error of the mean (s.e.m.). Transfer rates are given as the number of transconjugants per recipient cell. n = 4. Detection limit of the assay: 4.4×10^{-8} transconjugants per recipient.

Supplementary Table S4: Crystallographic data collection and refinement statistics for native $TraF_{1\text{-}194}$ (PDB: pdb_00008cba)

**$TraF\text{-}N_{1\text{-}194}$, PDB: pdb_00008cba**	
Data collection	
Wavelength [Å]	1.033
Resolution range [Å]	42.15 - 1.25 (1.30 - 1.25)
Space group	$P\ 2_1\ 2_1\ 2_1$
Unit cell	
a, b, c [Å]	48.88, 53.79, 83.23
α, β, γ [°]	90, 90, 90
Total reflections	682763 (62416)
Unique reflections	61401 (6018)
Multiplicity	11.1 (10.4)
Completeness [%]	99.94 (99.85)
Mean I/sigma(I)	14.15 (2.35)
Wilson B-factor [$Å^2$]	14.54
R-merge	0.0884 (0.853)
R-meas	0.0928 (0.898)
R-pim	0.0277 (0.277)
$CC_{1/2}$	0.996 (0.914)
CC*	0.999 (0.977)
Refinement	
Reflections used in refinement	61394 (6017)
Reflections used for R-free	2000 (197)
R-work	0.176 (0.289)
R-free	0.203 (0.310)
CC_{work}	0.955 (0.917)
CC_{free}	0.929 (0.938)
Number of non-H atoms	2043
macromolecules	1777
solvent	266
Protein residues	195
RMS_{bonds}	0.005
RMS_{angles}	0.78
Ramachandran	
Ramachandran favored [%]	98.45
Ramachandran allowed [%]	1.55
Ramachandran outliers [%]	0.00
Rotamer outliers [%]	0.99
Clashscore	2.52
Average B-factor [$Å^2$]	20.82
macromolecules	19.31
solvent	30.88

Statistics for the highest resolution shell are shown in parentheses

Supplementary Table S5 LFQ intensities and iBAQ values for each Tra protein acquired from TimsToF data of TraF pulldown before and after gel filtration. LFQ: Label free quantification, iBAQ: Intensity-Based Absolute Quantification.

**iBAQ**	**Pre-SEC**			**Post-SEC**		
Protein IDs	Run 1	Run 2	Run 3	Run 1	Run 2	Run 3
**TraB**	683030	476120	469240	641500	534360	641610
**TraC**	56163	67643	63803	95135	121570	57987
**TraD**	39217	20995	36249	28045	27179	71293
**TraE**	181670	178140	199220	198050	208750	219280
**TraF**	36006000	34155000	42259000	37068000	40427000	47165000
**TraG**	-	-	-	-	-	-
**TraH**	120000	95288	88118	142520	225150	115850
**TraI**	13671	11861	0	10041	40274	18478
**TraJ**	130620	137130	130260	255970	238400	236250
**TraK**	319660	384940	306370	763310	693990	755040
**TraL**	12473	12517	687.21	23723	19917	19967
**TraM**	632560	589020	604990	869520	908060	898120
**TraN**	-	-	-	-	-	-
**TraO**	90342	80764	102210	91227	87735	84387
**-: not found**						

**LFQ**	**Pre-SEC**			**Post-SEC**		
Protein IDs	Run 1	Run 2	Run 3	Run 1	Run 2	Run 3
**TraB**	2082300	1970200	1743100	2874000	2494800	2848000
**TraC**	0	409360	400240	400310	557140	0
**TraD**	166110	167320	187470	175830	188010	214600
**TraE**	1632500	1565800	1644100	1655700	1603500	1575300
**TraF**	86955000	86808000	84517000	105170000	97455000	100130000
**TraG**	-	-	-	-	-	-
**TraH**	833320	819660	558530	1899600	836700	975370
**TraI**	0	0	0	0	0	0
**TraJ**	880570	916410	892020	1496200	1517100	1469400
**TraK**	2000400	2258700	2165400	3773400	3724200	3725500
**TraL**	124020	0	0	272790	214980	258280
**TraM**	2680400	2617400	2658700	3959200	3887500	3963700
**TraN**	-	-	-	-	-	-
**TraO**	0	0	531960	406690	0	380670
**0: detectable but not quantifiable**<br>**-: not found**						

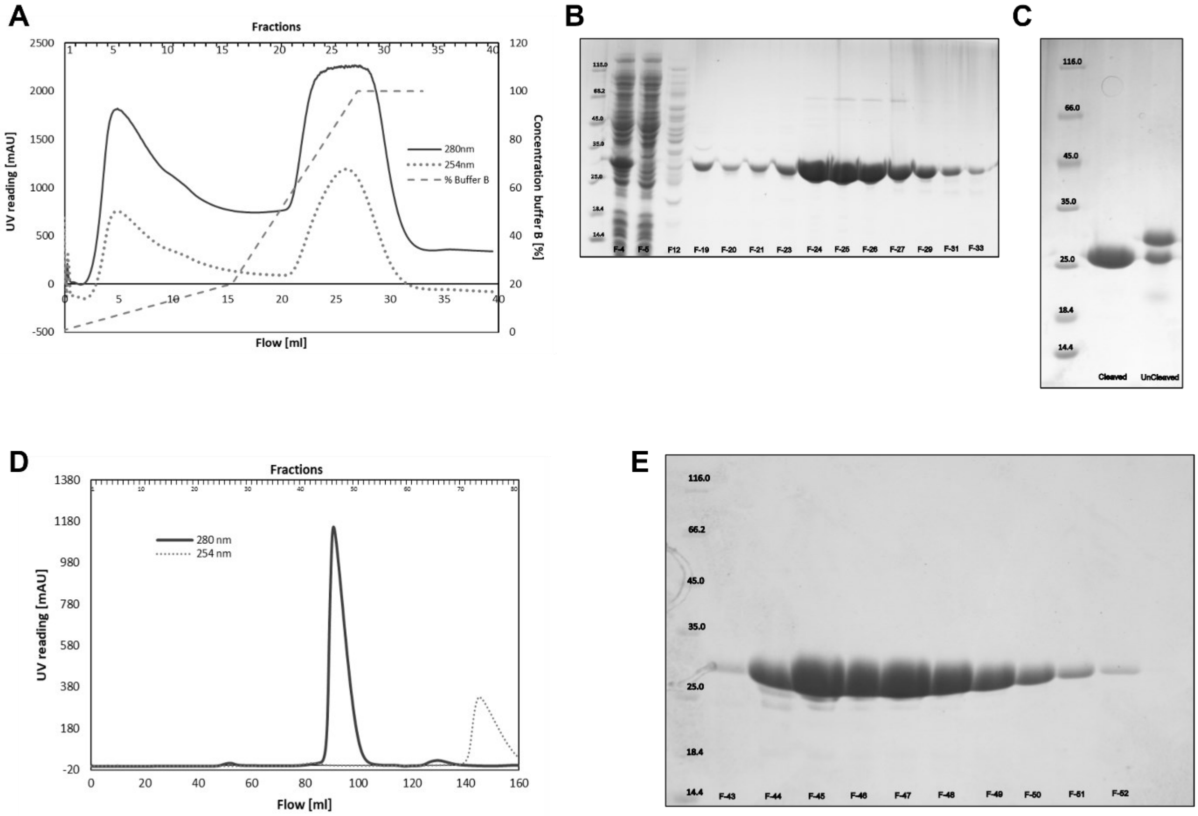

Supplementary Figure S1 Purification of TraF-N. (**A**): Chromatogram of TraF-N purification using a HisTrapFF column. Two distinct peaks were observed: the first represents impurities. TraF-N eluted in the second peak at higher imidazol concentration (buffer B) as shown in (**B**) analysis of the samples in a 12% SDS-PAGE. (**C**): 12% SDS-PAGE of the protein after TEV-cleavage. (**D**) UV chromatogram after size exclusion chromatography using an S200 16/60 GL column showing a single peak at 95 mL. (**E**): 12% SDS-PAGE of gel filtration elution fractions showing highly pure TraF-N protein, which was used in the experiments. Gel Marker: Pierce™ Unstained Protein MW Marker (Cat.: 26610).

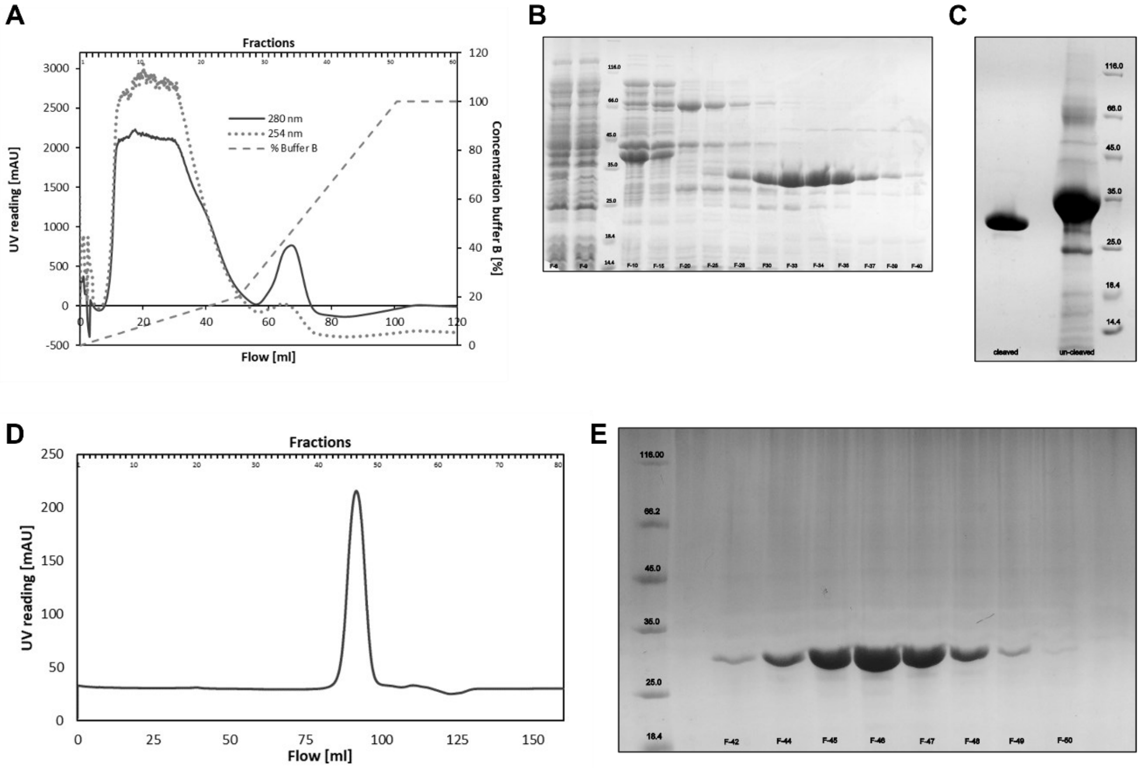

Supplementary Figure S2 Purification of TraF-C. (**a**) Chromatogram of TraF-C purification using a HisTrapFF column. Two distinct peaks were observed with the first representing impurities. At higher imidazole concentrations (buffer B), a small peak was observed corresponding to TraF-C as shown in (**b**) 12% SDS-PAGE of the samples. To further purify the protein, TEV-cleavage, followed by a second HisTrap purification, was performed. (**c**) 12% SDS-PAGE of the protein after TEV-cleavage. (**d**) UV chromatogram after size exclusion chromatography using an S200 16/60 GL column. A second purification step was carried out. **e)** 12% SDS-PAGE of the fractions obtained by size exclusion chromatography. A highly pure TraF-C was obtained, which was used in following experiments. Gel Marker: Pierce™ Unstained Protein MW Marker (Cat.: 26610).

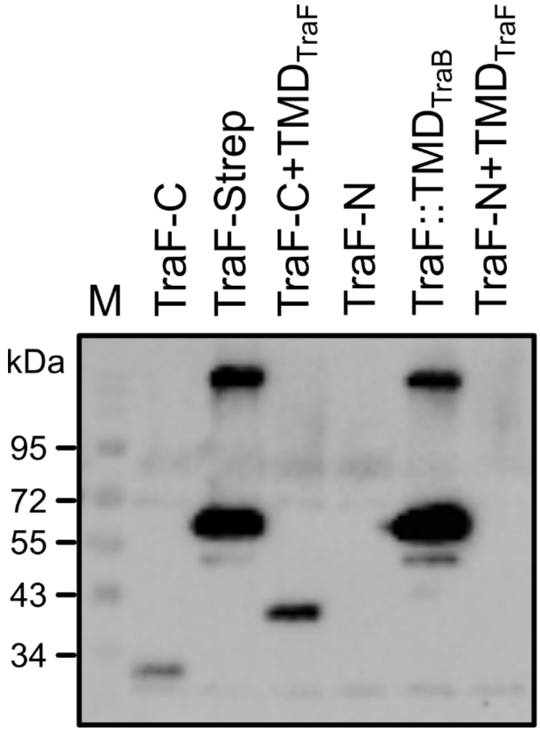

construct name	construct	kDa
TraF-C	TraF$_{239-450}$	24.4
TraF-Strep	TraF$_{1-450}$-Strep	53.8
TraF-C+TMD$_{TraF}$	TraF$_{176-450}$	31.8
TraF-N	TraF$_{1-212}$-Strep	26.6
TraF::TMD$_{TraB}$	TraF$_{1-214}$-TraB$_{45-64}$	52.7
TraF-N+TMD$_{TraF}$	TraF$_{1-238}$-Strep	29.3

Supplementary Figure S3 Immunoblot of TraF constructs expressed in *E. faecalis* JH2-2 used in biparental mating assays detected with anti-TraF antibody (raised against TraF C-terminal domain). Marker (M): Blue Prestained Protein Standard, Broad Range (11-250 kDa) (New England Biolabs, Ipswich, MA, USA).

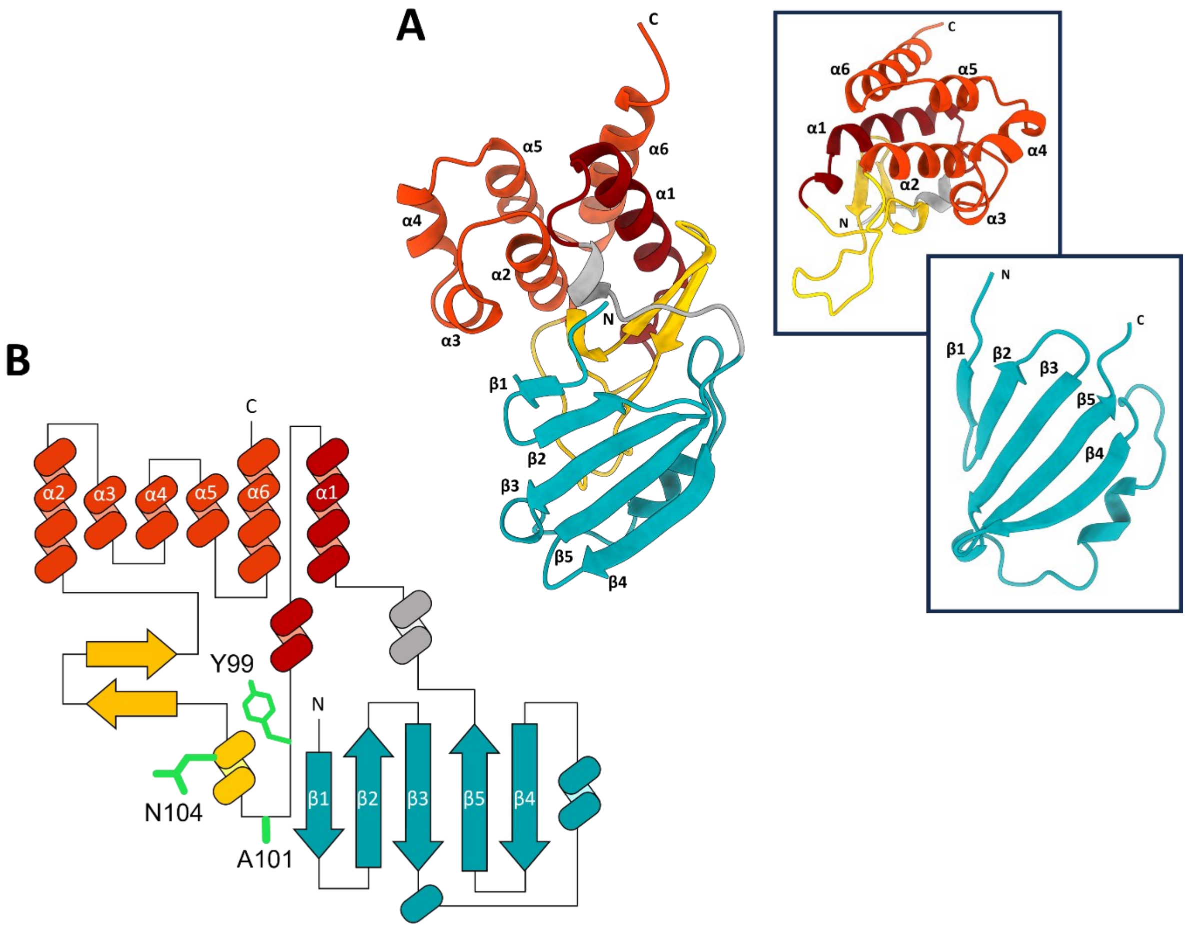

Supplementary Figure S4 Topology model of TraF-N. (**A**) Cartoon representation of the crystal structure of the N-terminal domain of TraF. N-terminal domain (β-sheet, blue): 1-63, C-terminal domain (α-helical, red): 132-194, additional α-helix (α1, dark red): 73-95, connecting loop between C-terminal domain and α1 (yellow): 96-131, connecting loop between α1 and N-terminal domain (grey): 64-72. (**B**) Topology view of TraF-N domains with highlighted mutated catalytic residues (green).

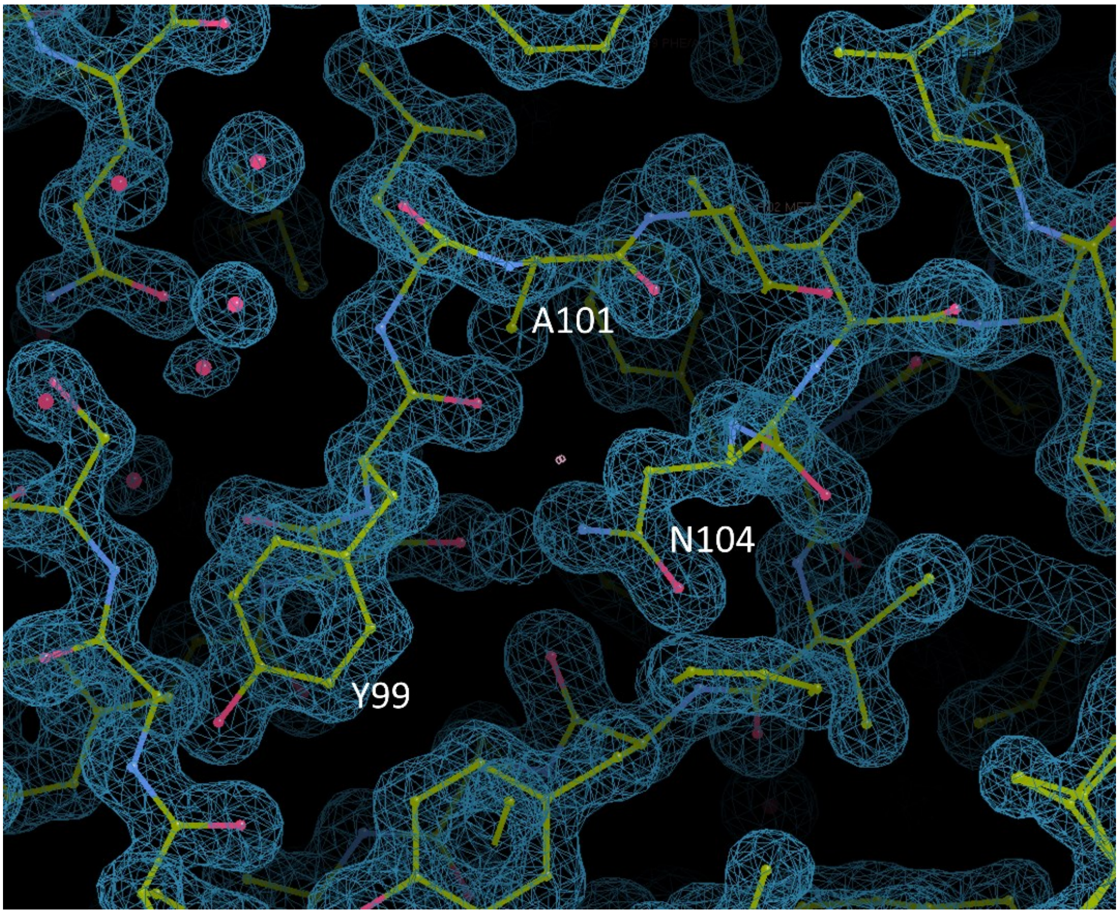

Supplementary Figure S5 Electron density ($2F_o$-F_c map) of TraF-N (pdb_00008cba). X-ray crystal structure at a contour level of 1.50 σ. Selected residues which represent the mutated catalytic triad are labelled.

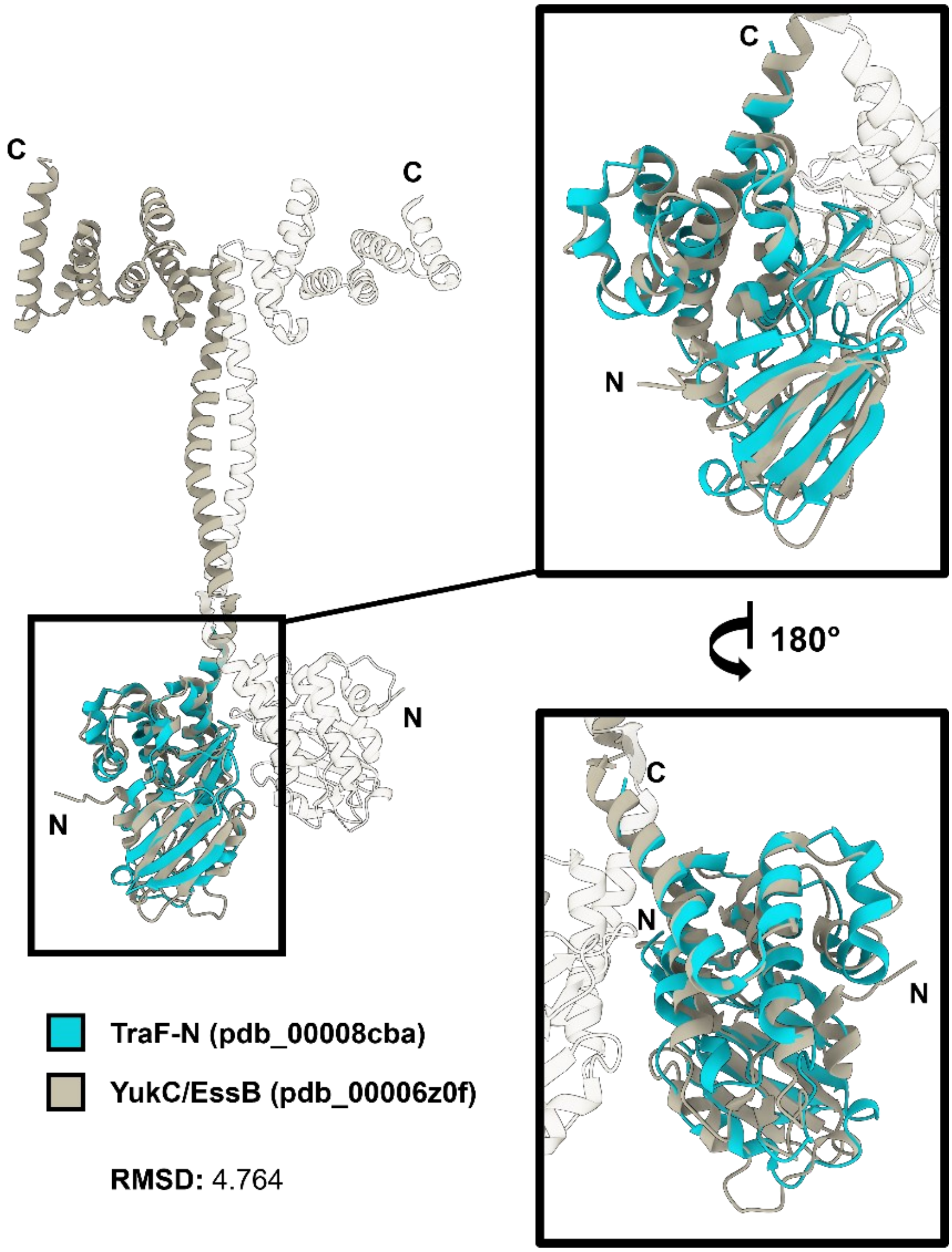

Supplementary Figure S6 Alignment of TraF-N crystal structure (pdb_00008cba, teal) to the crystal structure of YukC/EssB (pdb_00006z0f, tan). The RMSD was calculated using PyMOL cealign.

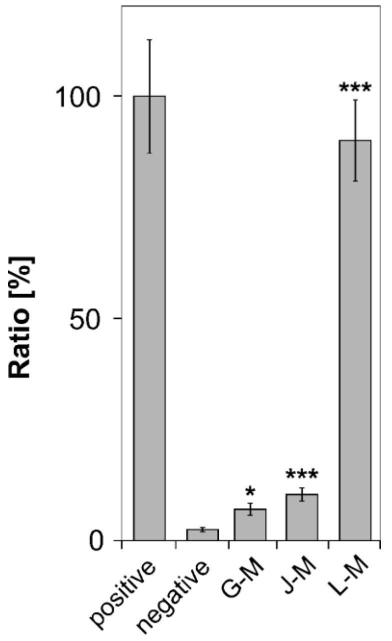

Supplementary Figure S7: B2H assay of $TraM_{B8}$ with pIP501 proteins, $TraG_{B1}$, $TraJ_{D4}$ and $TraL_{B6}$. A strong and statistically significant interaction was observed for $TraM_{B8}$ with $TraL_{B6}$. The positive control (GCN4 leucine zipper fused to the T18 and T25 fragments) was set to 100%. The other interactions are depicted as ratio to the positive control. As a negative control, the empty vectors were used. Error bars represent standard error of the mean (s.e.m.). *$p < 0.05$, ***$p < 0.001$.

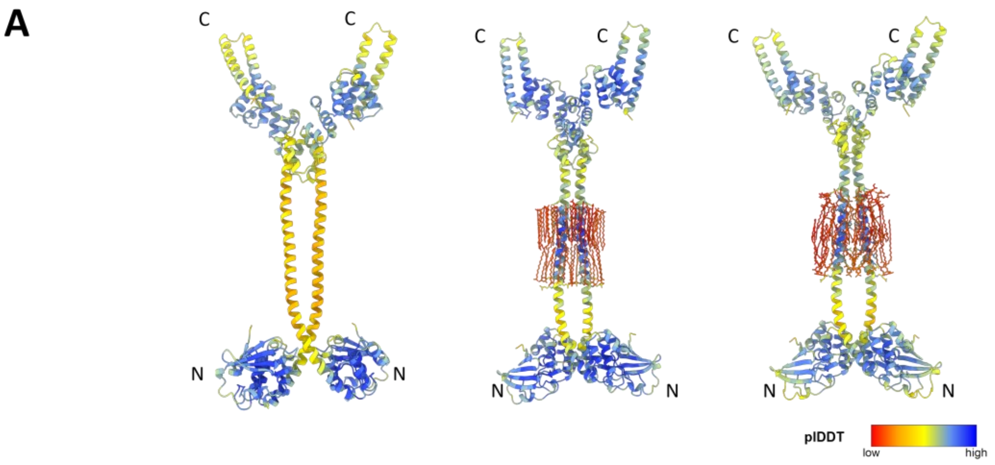

ligands	none	30 OLA + 30 PLM	15 PG + 5 CDL
pTM	0.39	0.50	0.54
ipTM	0.28	0.48	0.55

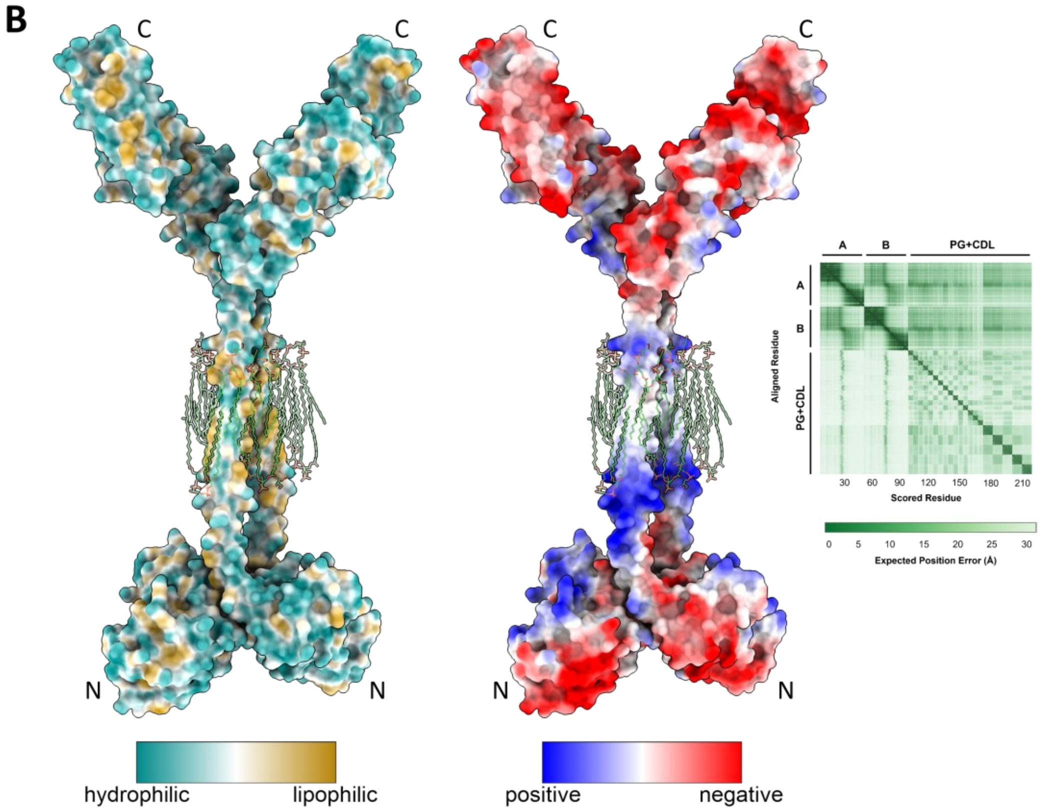

Supplementary Figure S8 (**A**) TraF dimer prediction with various membrane mimetics by AlphaFold3 coloured by per atom pIDDT score. Red: pIDDT<50, yellow: 70 > pIDDT > 50, light blue: 90 > pIDDT > 70, dark blue: pIDDT > 90. Higher values correspond to higher confidence that the model represents the true fold. OLA: oleic acid, PLM: palmitic acid, PG: phosphatidyl glycerol (PubChem CID: 44566653), CDL: cardiolipin (PubChem CID: 5287898, ePDB ID: CDL). A significant difference in pIDDT score is observed within the TMH domain. (**B**) TraF full length AlphaFold model with 15 PG and 5 CDL coloured by hydrophobicity and electrostatic potential with corresponding predicted alignment error (PAE) matrix.

A

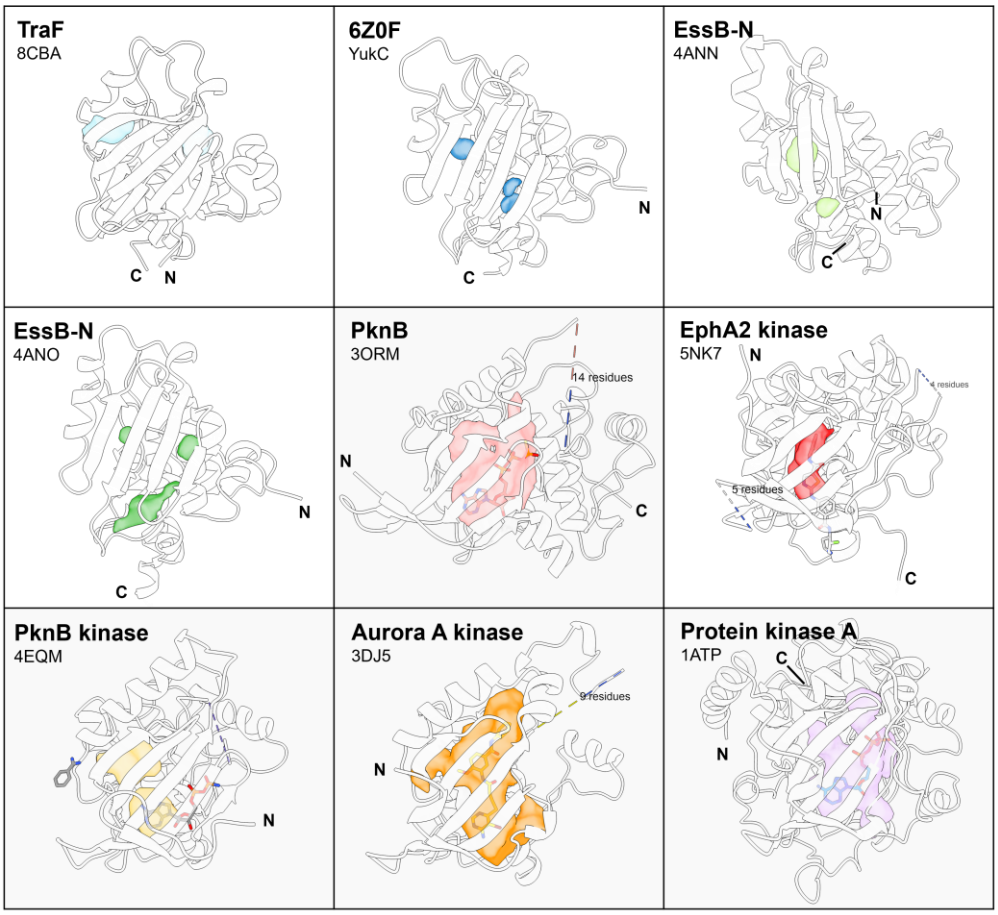

B

Structure		Origin organism	Catalytic residues			Activation	RMSD
			1	2	3	segment residue	to TraF-N
**TraF-N** (8CBA)		*E. faecalis*	Y99	A101	N104	Y117	target
**YukC** (6Z0F)		*B. subtilis*	I108	A110	N113	H126	3.337 (184)
**EssB-N** (4ANN)		*S. aureus*	V115	A117	E120	K132	3.389 (160)
**EssB-N** (4ANO)		*G. thermodenitrificans*	I106	C108	N111	H124	3.513 (184)
**PknB kinase** (3ORM)		*M. tuberculosis*	D138	K140	N143	D156	5.733 (160)
**EphA2 kinase** (5NK7)		*Homo sapiens*	D739	A741	N744	D757	6.391 (168)
**PknB kinase** (4EQM)		*S. aureus*	D132	K134	N138	D151	5.254 (160)
**Aurora A Kinase** (3DJ5)		*Mus musculus*	D269	K271	N274	D287	5.324 (152)
**Protein Kinase A** (1ATP)		*Mus musculus*	D166	K168	N171	D184	5.599 (184)

Supplementary Figure S9: Comparison of kinase and pseudokinase substrate cavities and catalytic triads. (**A**) Structures of identified pseudokinases and kinases were aligned by matchmaker (40% secondary structure similarity) and cavities identified by KVFinder accessed through ChimeraX (Guerra et al., 2024). The YukC (pdb_00006z0f) structure was truncated to the PK domain (residues 1-205) prior to alignment. Only chain A of PknB Kinase (pdb_00004eqm) was used. All structures are oriented in the same way looking onto the antiparallel β-sheet. (**B**) The structures were aligned using PyMOL (cealign) and the position of the residues was identified on structural level. Highlighted structures feature a fully functional catalytic triad (DxKxxN -12aa-D).

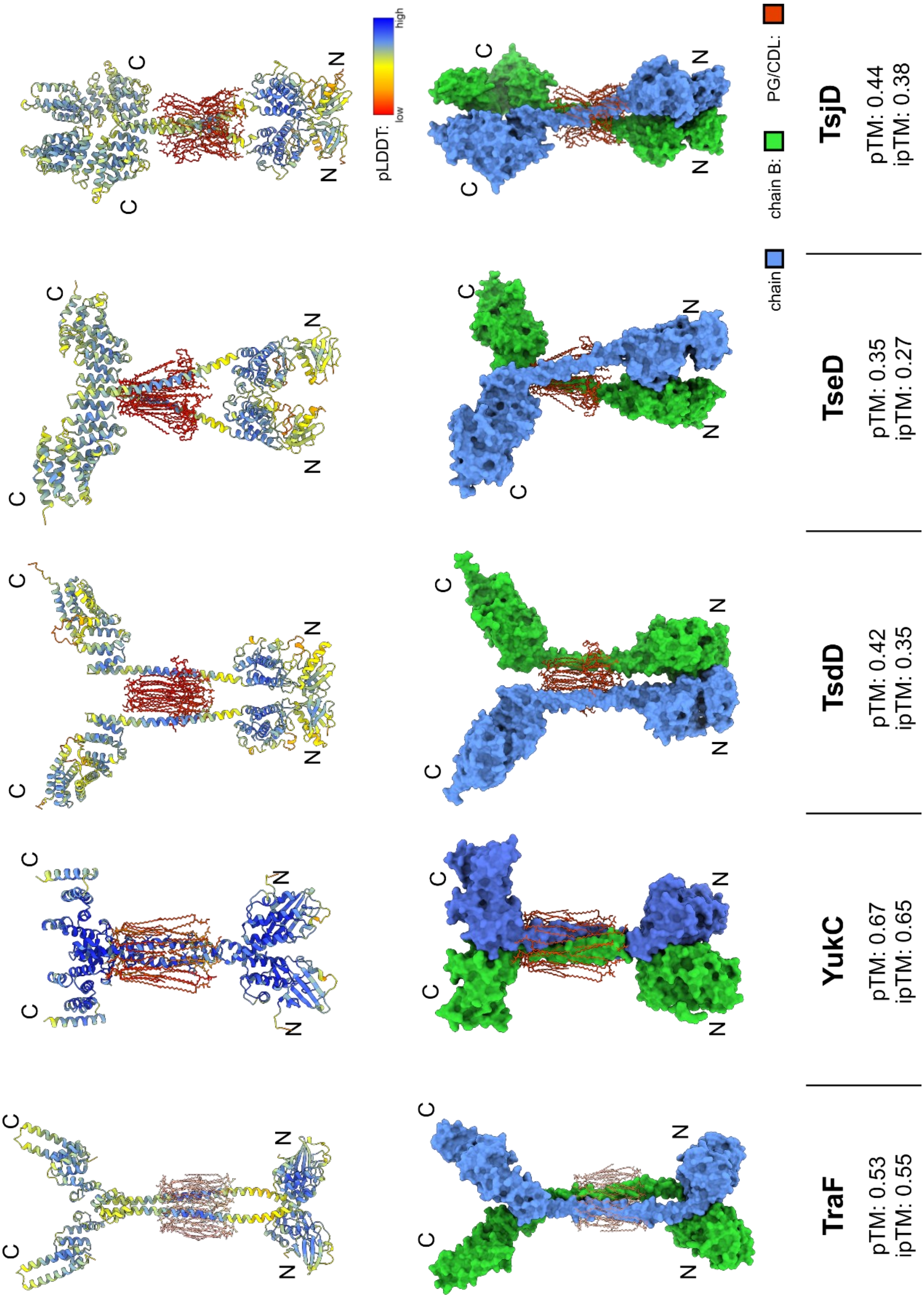

Supplementary Figure S10 Comparison of AlphaFold models from full length TraF with pseudokinases from various T7SSs: T7SSb (*S. aureus*, YukC), T7SSd (*Olsenella uli,* TsdD), T7SSe (*Bacillus sp.*, TseD) and T7SSj (*Clostridium felsineum*, TcjD). Upper models colored by plDDT score, lower models colored by chain. All structures apart from TscD show a distinct two domain structure with a transmembrane domain embedded in the predicted lipid patch. The N-terminal domain features a pseudokinase domain.

References

Arends K, Celik E-K, Probst I *et al.* TraG Encoded by the pIP501 Type IV Secretion System Is a Two-Domain Peptidoglycan-Degrading Enzyme Essential for Conjugative Transfer. *J Bacteriol* 2013;**195**:4436–44.

Berger TMI, Michaelis C, Probst I *et al.* Small Things Matter: The 11.6-kDa TraB Protein is Crucial for Antibiotic Resistance Transfer Among Enterococci. *Frontiers in Molecular Biosciences* 2022;**9**:314.

Eichenbaum Z, Federle MJ, Marra D *et al.* Use of the Lactococcal nisA Promoter To Regulate Gene Expression in Gram-Positive Bacteria: Comparison of Induction Level and Promoter Strength. *Applied and Environmental Microbiology* 1998;**64**:2763.

Ike Y, Craig RA, White BA *et al.* Modification of Streptococcus faecalis sex pheromones after acquisition of plasmid DNA. *Proc Natl Acad Sci USA* 1983;**80**:5369–73.

Jacob AE, Hobbs SJ. Conjugal Transfer of Plasmid-Borne Multiple Antibiotic Resistance in *Streptococcus faecalis* var. *zymogenes*. *J Bacteriol* 1974;**117**:360–72.

Karimova G, Pidoux J, Ullmann A *et al.* A bacterial two-hybrid system based on a reconstituted signal transduction pathway. *Proc Natl Acad Sci USA* 1998;**95**:5752–6.

Kohler V, Goessweiner-Mohr N, Aufschnaiter A *et al.* TRAN: A novel repressor of an Enterococcus conjugative type IV secretion system. *Nucleic Acids Research* 2018;**46**:9201–19.

Leenhouts K, Buist G, Bolhuis A *et al.* A general system for generating unlabelled gene replacements in bacterial chromosomes. *Mol Gen Genet* 1996;**253**:217–24.

Scheich C, Niesen FH, Seckler R *et al.* An automated in vitro protein folding screen applied to a human dynactin subunit. *Protein Science* 2004;**13**:370–80.
